# Stress granules are dispensable for mRNA stabilization during cellular stress

**DOI:** 10.1093/nar/gku1275

**Published:** 2014-12-08

**Authors:** Nadine Bley, Marcell Lederer, Birgit Pfalz, Claudia Reinke, Tommy Fuchs, Markus Glaß, Birgit Möller, Stefan Hüttelmaier

**Affiliations:** 1 Division of Molecular Cell Biology, Institute of Molecular Medicine, Martin Luther University Halle-Wittenberg, Heinrich-Damerow-Strasse 1, 06120 Halle, Germany; 2 Core Facility Imaging (CFI) of the Medical Faculty, Institute of Molecular Medicine, Martin Luther University Halle-Wittenberg, Heinrich-Damerow-Strasse 1, 06120 Halle, Germany; 3 Genome Biology Unit, European Molecular Biology Laboratory, Meyerhofstrasse 1, 69117 Heidelberg, Germany; 4 Institute of Computer Science, Martin Luther University Halle-Wittenberg, Von-Seckendorff-Platz 1, 06099 Halle, Germany

## Abstract

During cellular stress, protein synthesis is severely reduced and bulk mRNA is recruited to stress granules (SGs). Previously, we showed that the SG-recruited IGF2 mRNA-binding protein 1 (IGF2BP1) interferes with target mRNA degradation during cellular stress. Whether this requires the formation of SGs remained elusive. Here, we demonstrate that the sustained inhibition of visible SGs requires the concomitant knockdown of TIA1, TIAR and G3BP1. FRAP and photo-conversion studies, however, indicate that these proteins only transiently associate with SGs. This suggests that instead of forming a rigid scaffold for mRNP recruitment, TIA proteins and G3BP1 promote SG-formation by constantly replenishing mRNPs. In contrast, RNA-binding proteins like IGF2BP1 or HUR, which are dispensable for SG-assembly, are stably associated with SGs and the IGF2BP1/HUR-G3BP1 association is increased during stress. The depletion of IGF2BP1 enhances the degradation of target mRNAs irrespective of inhibiting SG-formation, whereas the turnover of bulk mRNA remains unaffected when SG-formation is impaired. Together these findings indicate that the stabilization of mRNAs during cellular stress is facilitated by the formation of stable mRNPs, which are recruited to SGs by TIA proteins and/or G3BP1. Importantly, however, the aggregation of mRNPs to visible SGs is dispensable for preventing mRNA degradation.

## INTRODUCTION

In response to environmental stress or infection, bulk protein synthesis is severely reduced due to the impairment of the initiation step of mRNA translation. This is mainly facilitated by the stress-dependent activation of kinases phosphorylating the translation initiation factor eIF2α (EIF2S1). The phosphorylation of eIF2α impairs the assembly of the ternary eIF2/tRNA_i_^Met^/GTP complex, which is essential for the initiation of mRNA translation and thus results in the stalling of bulk mRNA in 48S ‘pre-initiation’ complexes (1–3). These associate with various RNA-binding proteins (RBPs) in cytoplasmic mRNPs, which transiently assemble into cytoplasmic stress granules (SGs). This stress-induced assembly or aggregation of mRNPs was suggested to essentially rely on the aggregation of RBPs comprising prion-like or low complexity (LC) regions, which are frequently observed in intrinsically disordered (ID) proteins [reviewed in (4,5)]. In accord, suggested ID-like RBPs including TIA1, TIAR (TIAL1) or G3BPs were demonstrated to induce the formation of SG-like cytoplasmic granules at high cytoplasmic concentration, for instance, when transiently overexpressed in tissue cultured cells (6,7). *In vitro*, some of the ID-like proteins found in SGs were shown to induce the formation of hydrogels, which resemble RNA germ cell granules in *Caenorhabditis elegans* (8). The latter behave like liquid droplets formed or maintained by the transient interaction of LC-containing RBPs, which presumably associate with mRNPs containing translationally stalled mRNAs. Like in germ cells, the dynamic association of LC-containing RBPs and mRNPs in stressed cells was proposed to induce a ‘demixing phase transition’ resulting in the formation of hydrogel-like cytoplasmic SGs [reviewed in (4,5)]. Recent studies propose that the increase of non-polysome associated mRNA during cellular stress is essential and sufficient to trigger the formation of SGs (9).

Although various SG-recruited proteins have been identified to date, the physiological role of SGs remains largely elusive. Initially, it was proposed that SGs are essential to facilitate the block of bulk mRNA translation during cellular stress (1). However, even when the formation of SGs visible by fluorescence microscopy was impaired by the depletion of factors essential for their assembly, the stress-induced inhibition of bulk protein synthesis appeared largely unaffected (10,11). Moreover, recent studies have indicated that SGs disassemble before mRNA translation is fully restored in cells recovering from cold shock (12). These findings strongly suggest that the block of mRNA translation observed during cellular stress can be facilitated in a largely SG-independent manner. Despite a potential involvement of SGs in reorganizing the ‘stress-translatome’, it was proposed that the formation of SGs promotes cell viability (13). However, it has to be noted that the vast majority of studies addressing the biological role of SGs rely on the depletion of factors proposed to be essential for the formation of these foci. These factors are likely to serve SG-independent roles in enhancing cell viability and thus their depletion may impair cellular fitness in a largely SG-independent manner.

Various recent findings suggest that SGs serve an essential role in modulating intracellular signaling during cellular stress. This view is supported by the SG-recruitment of various key signaling factors and the observation that perturbing specific signaling cascades impairs the formation of SGs, respectively. For instance, it was demonstrated that RSK2 (p90 ribosomal S6 kinase) depletion interferes with the formation of SGs and reduces cell survival in response to cellular stress (14). Notably, RSK2 is recruited to SGs by associating with the putative prion-like C-terminus of TIA1, a proposed key factor in the assembly of SGs. The recruitment of RSK2 to SGs was suggested to prevent or ‘slow down’ the induction of apoptosis during cellular stress. Similar mechanisms were also proposed for the stress-induced recruitment of RACK1 or ROCK1 to SGs. The SG-facilitated sequestration of these factors was associated with reduced activation of JNK or JIP-1 dependent apoptosis, respectively [reviewed in (4)]. Notably in this respect, the depletion of O-GlcNac transferase, which modifies various cellular proteins including key signaling factors like RACK1, was shown to impair the formation of SGs (10). Consistent with a role of SGs in modulating the ROCK1-facilitated control of apoptosis, it was proposed that the Wnt-dependent activation of Rac1 interferes with the RhoA-dependent activation of SG-assembly [reviewed in (4)]. Most recently, it was revealed that the inhibition of mTORC1 by the sequestering of RAPTOR in SGs prevents or severely limits the apoptosis of cancer cells during cellular stress (15). In summary, it appears tempting to speculate that the assembly of SGs interconnects the stress-induced block of bulk mRNA translation and key signaling cascades modulating cell viability and apoptosis [reviewed in (4)].

In previous studies, we demonstrated that the IGF2 mRNA binding protein 1 (IGF2BP1), also termed ZBP1 (Zipcode binding protein 1), is recruited to SGs together with its target transcripts (16,17). This transient enrichment in SGs was correlated with the selective stabilization of IGF2BP1-target mRNAs including MYC, ACTB and MAPK4 (16,18). These findings prompted us to speculate that the selective stabilization of target mRNAs during cellular stress allows the identification of novel RBP-target transcripts. Accordingly, we analyzed if the transient depletion of IGF2BP1 allows the identification of target mRNAs in stressed tumor-derived cells. As expected, we identified almost a hundred novel target candidate transcripts, which were selectively decreased in stressed cells upon the knockdown of IGF2BP1 (18). Notably, these target mRNAs are also subjected to IGF2BP1-dependent regulation of mRNA translation and/or turnover in non-stressed cells (18,19). Hence, the selective stabilization of mRNAs by RBPs during cellular stress allows the identification of target mRNAs regulated by the same proteins, presumably via cytoplasmic mRNPs (20), also in non-stressed cells. However, it remained elusive if the selective stabilization of mRNAs observed during cellular stress is facilitated via SGs.

In this study, we addressed the role of SGs in the stress-induced stabilization of mRNAs. To this end we aimed at establishing protocols allowing the impairment of SG-formation without perturbing stress signaling. In remarkable contrast to various previous reports, our studies reveal that the sustained inhibition of SGs visible by fluorescence microscopy requires the concomitant depletion of TIA proteins and G3BP1. FRAP and photo-conversion studies, however, indicate that these proteins rapidly shuttle between SGs and the cytoplasm with barely any immobile fraction in SGs. The opposite is observed for RBPs dispensable for SG-formation including IGF2BP1, YB1 (YBX1) or HUR (ELAVL1). This provides further evidence that the recruitment of mRNPs into SGs by TIA proteins and G3BPs relies on their transient association/aggregation instead of forming a rigid and largely non-dynamic scaffold for mRNP-association. Finally, our studies reveal that the stabilization of bulk mRNA including IGF2BP1 target transcripts is largely independent of SG-formation. Taken together these findings provided strong evidence that the aggregation of mRNPs in SGs neither required for the control of bulk mRNA translation nor the stabilization of translationally stalled transcripts.

## MATERIALS AND METHODS

### Cell culture, transfections and treatments

U2OS, HUH7 and HEK293 cells were cultured in Dulbecco's modified Eagle's medium supplemented with 10% fetal bovine serum (FBS). Where stated, plasmids (48 h) or siRNAs (72 h) were transfected, according to manufacturer's instructions, using Lipofectamine 2000 or RNAiMax (Life Technologies), respectively. Cells were transduced by lentiviral vectors, as previously described (18,19). All siRNAs and plasmids are depicted in Supplementary Table S1. All transfected cells were splitted 24 h post-transfection to allow application of different assays from the same transfection and/or the analysis or several (stress) conditions. Oxidative or endoplasmic reticulum (ER)-stress was induced using sodium-arsenate (2.5 mM) or thapsigargin (1 μM) for indicated time. Actinomycin D (ActD) was added (5 μM) to block transcription for the time indicated.

### Western blotting

Western blotting was essentially performed as recently described using RIPA-buffer (20 mM Tris-HCl (pH 7.5), 150 mM NaCl, 1 mM EDTA, 1 mM EGTA, 1% NP-40, 1% sodium deoxycholate (DOC), 2.5 mM sodium pyrophosphate, 1 mM beta-glycerophosphate, 1 mM Na_3_VO_4_; supplemented with protease inhibitor cocktail (Sigma Aldrich)) to lyse cells (18,19). For used primary antibodies please refer to Supplementary Table S2. All secondary antibodies used were described before (18).

### Co-immunopurification

Cells were lysed in gradient buffer (10 mM Hepes pH7.4, 150 mM KCl, 5 mM MgCl_2_, 0.5% NP40; supplemented with protease and phosphatase inhibitors). Proteins were immune-purified by indicated antibodies immobilized to Dynabeads® Protein G (Life Technologies). After intense washing, proteins were eluted using sodium dodecyl sulphate sample buffer and analyzed by western blotting, essentially as described previously (17).

### Metabolic labeling by ^35^S-methionine

U2OS cells transfected with indicated siRNAs were pre-incubated with methionine-free medium overnight 48 h post-transfection. Protein *de novo* synthesis was monitored by the addition of 100 μCi ^35^S methionine per 6-well for 1 h. Where indicated cells were simultaneously stressed by arsenate. Upon extensive washing cells were extracted in RIPA buffer. Equal amounts of total protein were analyzed by western blotting using Ponceau-staining to analyze protein loading and phosphor-imaging to determine newly synthesized proteins.

### Sucrose gradient centrifugation

U2OS cells (∼1.5 Mio cells) transfected with indicated siRNAs and stressed by arsenate when stated were lysed in gradient buffer 72 h post-transfection. Total protein (D_C_ protein assay, Bio-Rad) and RNA (OD_260_) concentrations were determined to ensure equal loading of the gradients. Linear 15–45% sucrose (w/v) gradients in gradient buffer lacking NP-40 were centrifuged in a Beckman SW-40 rotor at 30 000 revolutions per minute for 2 h. Gradients were fractionated and ultraviolet-profiles were monitored by a Foxy Jr. fraction collector (Teledyne) with syringe pump (Brandel) (18).

### Quantitative reverse transcriptase-polymerase chain reaction (qRT-PCR) and microarray analyses

Changes in RNA abundance were determined by qRT-PCR as recently described (18,19). All gene-specific primer pairs used are shown in Supplementary Table S1. For microarray analyses, total RNA was extracted using TRIZOL and further purified using RNeasy MinElute Cleanup Kit (QIAGEN). RNA integrity and concentration was then examined on an Agilent 2100 Bioanalyzer (Agilent Technologies, Palo Alto, CA, USA) using the RNA 6.000 LabChip Kit (Agilent Technologies). Array analyses were performed at the microarray core facility of the IZKF (Leipzig, Germany) essentially as recently described using two independent chip systems (Affimetrix and Illumina) (18). For the analysis with Affimetrix HG133plus 2.0 chips U2OS cells were transfected with control (siC) siRNAs or siRNAs directed against TIA-1, TIAR and G3BP1 (siSGs). Where indicated cells were treated with arsenate and ActD for 2 h (stress). All samples were analyzed in duplicates. Raw, Mas5- or RMA-normalized data of Affimetrix chips were analyzed using Bioconductor (www.bioconductor.org) and R (www.r-project.org). Reliably detected transcripts in both untreated samples identified by Mas5 present/absent calls were further analyzed. Ratios (knockdown versus control) of log2 expression data from non-stressed cells were plotted against the corresponding ratios of stressed cells to determine stress- and knockdown-dependent changes in RNA abundance.

For the analysis using Solexa HumanHT-12 chips (Illumina) U2OS cells were transfected with two independent sets of TIA-1, TIAR and G3BP1-directed (siSGs) or control (siC) siRNAs and treated with arsenate and ActD for indicated time. Reliably detected transcripts in all untreated samples were identified by a *P*-value less than 0.001. A linear regression was applied to the quantile-normalized and background corrected non-logarithmic expression data to determine the mRNA degradation over time (slope) for siSGs versus siC transfected cells. Pearson's correlation analyses were used to determine how the inhibition of SG-formation affects bulk mRNA degradation.

### Microscopy and image analyses

Indirect immunostaining was essentially performed as previously described (16). For primary antibodies please refer to Supplementary Table S2. All secondary antibodies were previously described (18). Images were acquired on a Leica TCS-SP5X CLSM equipped with a Ludin live chamber or a Nikon TE-2000E fluorescence microscope using 63× magnification and standardized settings. The area fraction representing the number and size of SGs was automatically quantified using the Mica2D particle detector of MiToBo (www.informatik.uni-halle.de/mitobo), an extension package for ImageJ (www.imagej.nih.gov/ij/). To allow an assessment of SG parameters for individual cells the cell area was manually labeled.

For FRAP and photo-conversion analyses, U2OS cells were transiently transfected for 24 h before seeding on glass bottom dishes (MatTek). Where indicated stably expressing cell clones were generated by G418 and at least two distinct clones were included in the studies. If not indicated otherwise, cells were stressed by arsenate for 25 min. Imaging was conducted up to 1 h after arsenate treatment. FRAP analyses were performed on the TCS-SP5X using the provided FRAP wizard. The region of interest was selected to cover a single SG and fluorescence was bleached using the Argon laser (488 nm) at maximal power. Photo-conversion analyses were performed using the FRAP wizard using standard settings for the concomitant detection of green-fluorescent protein (GFP) (Argon laser: 488 nm) and red-fluorescent protein (RFP) (DPSS laser: 561 nm) fluorescence. Photo-conversion was induced by using the bleach point function of the LAS AF software package at maximal laser power (Argon laser: 488 nm) for 100 ms. The bleach point was chosen in close proximity to a single SG without affecting additional SGs. For both, FRAP as well as photo-conversion analyses, the recovery or loss of fluorescence signal was recorded at a 300 ms time interval for five frames before and 100 or 300 frames after bleaching or conversion, respectively. The wizard software application was used to normalize the fluorescence intensities for background bleaching or conversion. The traffic model for molecules moving in and out of SGs can be described by first-order kinetics, which are *k*_1_*M*_IN_ = d*M_IN_*/d*T* and *k*_2_*M*_OUT_ = d*M*_OUT_/d*T*. *k*_1_*M*_IN_ and *k*_2_*M*_OUT_ describe the traffic constants, whereas *M*_IN_ and *M*_OUT_ are the number of molecules moving into and out of SGs. At steady-state levels incoming and outgoing molecules are expected to be balanced and thus the corresponding half-lives are considered to be *t*_1IN_ = *t*_2OUT_. FRAP parameters determined by first-order kinetics are summarized in Supplementary Figure S8A.

## RESULTS

### Inhibiting SG-formation without affecting stress signaling

To analyze whether the IGF2BP1-dependent stabilization of target mRNAs during cellular stress is essentially facilitated via SGs, we aimed at inhibiting or severely reducing SG formation without affecting the phosphorylation of eIF2α or the block of bulk mRNA translation (1,3). Initially, we focused our efforts on approaches previously reported to impair the assembly of SGs visible by fluorescence microscopy. These included: (i) the knockdown of FMRP (21); (ii) the transient depletion of ataxin-2 (22); (iii) the knockdown of RSK2 (14); (iv) the forced expression of TIA1-ΔRRM (3,6); (v) the depletion of HDAC6 (23); (vi) the knockdown of TIA-proteins and/or G3BP1, since these proteins were suggested as key factors of SG assembly (6,24). For all these approaches, SG formation was monitored in U2OS and/or Huh7 cells upon the transient depletion or overexpression of the respective protein factors. Initially, stress was induced by 1 h of arsenate treatment and SG formation was monitored by fluorescence microscopy upon immunostaining of key SG-components including IGF2BP1, YB1, TIA proteins and/or G3BP1, respectively. The number of SG-positive cells as well as the SG-area fraction indicating the percentage of cell area covered by SGs was determined manually. In the following we refer to SGs when mRNP-aggregates were detectable by fluorescence microscopy.

**Figure 1.**
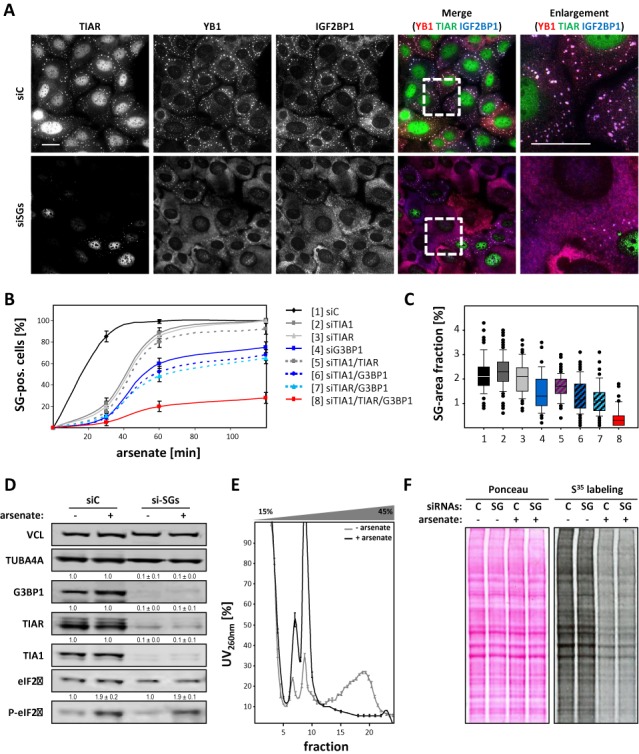
The concomitant depletion of TIA proteins and G3BP1 impairs SG formation. (**A**) U2OS cells transfected with indicated siRNAs (siC, control; siSG, siTIA1, siTIAR and siG3BP1) for 72 h were stressed by arsenate (2.5 μM) for 2 h before immunostaining of indicated proteins. Enlargements of the boxed regions in the merged images are shown in the right panel. Bars, 25 μm. (**B** and **C**) The average number of SG containing cells (B) and the SG area fraction (C) was analyzed by immunostaining for IGF2BP1 and YB1 in U2OS cells transfected with indicated siRNAs. Both parameters were determined by an automated particle detection tool, adapted from (18), after indicated times of arsenate stress. Error bars indicate SD determined by analyzing at least 100 cells per condition in three independent experiments. (**D**) The phosphorylation of eIF2α in non-stressed (−) or arsenate (+, as in A) stressed U2OS cells transfected with control (siC) or siSG (as in A) siRNAs was determined by western blotting with indicated antibodies. VCL and TUB4A4 served as loading controls to determine knockdown efficiencies as indicated by numbers above each panel. Standard deviation was determined from three independent experiments. (**E**) The association of bulk (m)RNA with polysomes was monitored by linear (15–45% w/v) sucrose gradient centrifugation in stressed (+, arsenate) versus non-stressed (−, arsenate) U2OS cells transfected with control (siC) or siSG siRNAs, as in (A). The distribution of RNA was monitored by UV spectroscopy and is shown as the average absorbance determined for individual fractions isolated from siC- and siSG-transfected samples. Error bars indicate SD determined in three independent studies for siC- and siSG-transfected cells. (**F**) Protein synthesis in arsenate-stressed (+) versus non-stressed (−) U2OS cells transfected as in (A) was analyzed by metabolic labeling using S^35^-methionine. The fraction of newly synthesized proteins was determined by western blotting using autoradiography (right panel). Equal loading was controlled by Ponceau staining (left panel).

The role of fragile X mental retardation protein (FMRP) in SG formation was monitored in tumor-derived U2OS cells upon the siRNA-directed depletion of FMRP (Supplementary Figure S1A and B). Although the endogenous FMRP was recruited to SGs, SG assembly induced by arsenate remained largely unchanged by the knockdown of FMRP (Supplementary Figure S1A and B). To exclude bias due to knockdown efficiencies or the transfection procedure we extended our analyses to immortalized FMRP (-/-) mouse embryonic fibroblasts (MEFs) versus KO-MEFs stably transduced with Flag-tagged FMRP (25). As in U2OS cells, the exogenous FMRP was recruited to SGs, but SG assembly induced either by arsenate (1 h), arsenite (30 min) or thapsigargin (1 h) (data not shown for arsenite and thapsigargin) appeared largely unaffected by the loss of FMRP (Supplementary Figure S1C and D). Likewise, the number of SG-positive U2OS or Huh7 cells remained essentially unchanged upon the knockdown of ataxin-2 (Supplementary Figure S2A–D). Surprisingly, in Huh7 SG formation appeared even enhanced upon ataxin-2 depletion as evidenced by an increase in the apparent size of SGs. The transient depletion of RSK2 impaired the arsenate-dependent formation of SGs as suggested (data not shown). However, this inhibition was correlated with an impaired arsenate- or thapsigargin-induced phosphorylation of eIF2α (Supplementary Figure S3A). This confirmed previous reports indicating RSK2 to modulate the activation of PKR and thus the stress-dependent activation of eIF2α (26,27). TIA1 is a key marker of SGs, which was shown to facilitate SG-formation via a C-terminal prion-like domain (3,6). However, upon deletion of the N-terminal RRM-domains, the truncated TIA1-ΔRRM protein was proposed to impair the formation of SGs when transiently expressed in tissue-cultured cells. As reported, TIA1-ΔRRM appeared to impair the formation of ‘regular’ IGF2BP1-containing SGs in ∼45% of transiently transfected cells (Supplementary Figure S3B). However, in the majority of cells, ‘regular’ SGs were formed. Surprisingly, these also contained TIA1-ΔRRM. Most notably, however, we also observed an impairment of SG formation in ∼20% of GFP-transfected cells and in cells expressing high levels of IGF2BP1, HUR or YB1 (data not shown). Consistent with recent studies (9), this suggested that the transient expression of proteins, in particular RBPs, can interfere with the formation of SGs (Supplementary Figure S3B). The knockout of the SG-recruited histone deacetylase 6 (HDAC6) was reported to prevent the formation of SGs in stressed MEFs (23). However, in U2OS cells we failed to confirm the recruitment of endogenous as well as GFP-tagged HDAC6 to thapsigargin- (data not shown) or arsenate-induced SGs (Supplementary Figure S4A and B). Moreover, the assembly of SGs was unchanged in cells depleted of HDAC6 suggesting that SG-formation is largely uncoupled from HDAC6 function in cancer-derived cells (Supplementary Figure S4C and D). Finally, we investigated if the formation of SGs can be inhibited by the depletion of TIA proteins and/or G3BP1. In previous studies, it was demonstrated that the formation of SGs is significantly impaired in TIA1 KO-MEFs stressed by arsenite for 30 min (6). Moreover, it was proposed that the G3BP1 protein is an essential facilitator of SG formation (7). However, the assembly of SGs in U2OS cells treated with arsenate for 1 h was only modestly decreased by the depletion of TIA1 or TIAR (Supplementary Figure S5A–D). This supported recent findings suggesting TIA1 only plays a minor role in SG formation (9). In cells transfected with G3BP1-directed siRNAs, the impairment of SG-formation was significantly more pronounced with ∼40% of cells lacking visible SGs after 1 h of arsenate treatment. Consistently, the ‘area fraction of SGs’ was only modestly reduced by the knockdown of TIA1 or TIAR yet significantly decreased by the depletion of G3BP1. However, the depletion of individual factors appeared insufficient for severely inhibiting SG formation, as previously suggested for G3BP1/2 depletion (28). This suggested that the concomitant depletion of these factors enhances the impairment of SG formation even at late time points of stress application. To evaluate this by quantitative means and in a time-resolved manner, we adapted an automated tracing algorithm previously used for identifying focal contacts (18). This allowed the automated tracing of SGs labeled by indirect immunostaining of YB1 and IGF2BP1 in U2OS cells treated with arsenate for 30–120 min (Figure 1A–C). In U2OS cells transfected with control siRNAs (siC), ∼80% of cells contained SGs already 30 min after arsenate application (Figure 1B). Consistent with previous studies (6), the depletion of TIA1 or TIAR essentially abolished SG formation after 30 min of stress induction but ∼80% of cells contained SGs after 1 h of arsenate treatment. This delay of SG assembly was significantly enhanced upon the depletion of G3BP1 with only 70% of SG-positive cells after 2 h of arsenate treatment. The concomitant knockdown of all three factors in U2OS, termed SG-knockdown/-depletion (siSGs, SGD), severely impaired SG formation and reduced the number of SG-positive cells to ∼20% of control levels after 2 h of arsenate-induced stress (Figure 1A and B). In agreement with a severely reduced assembly of SGs, we observed a striking reduction in the SG-area fraction upon the depletion of all three proteins (Figure 1C). This was confirmed by an additional set of siRNAs, in another cell line (Huh7) and thapsigargin to test an additional stressor (Supplementary Figure S6A–C). As observed in U2OS cells, SG formation was essentially abolished by the concomitant depletion of TIA1, TIAR and G3BP1 irrespective of the used stressor or cell line. To exclude that the impairment of SG formation resulted from aberrant stress signaling, the phosphorylation of eIF2α was monitored upon the SGD (Figure 1D; Supplementary Figure S6B). In both cell lines analyzed, U2OS as well as Huh7, the upregulation of eIF2α phosphorylation by arsenate or thapsigargin appeared unchanged by the SGD suggesting that ‘stress signaling’ was largely unaffected. Consistently, sucrose gradient centrifugation revealed that the depletion of polysomes and the shift of bulk (m)RNA to pre-polysomal fractions was preserved in arsenate-stressed cells when SG assembly was abolished by the SGD (Figure 1E). UV260 reads of indicated fractions were averaged over cell populations transfected with control or TIA1-/TIAR-/G3BP1-directed siRNAs. Thus, the small variation in UV reads indicated that the SGD neither affected mRNA translation in non-stressed (Figure 1E, gray) nor the block of bulk mRNA translation in arsenate-stressed cells (Figure 1E, black). Finally, this was evaluated by the metabolic labeling of stressed (+, arsenate) or non-stressed (−, arsenate) cells transfected with control (C) or TIA1-/TIAR-/G3BP1-directed (SG) siRNAs (Figure 1F). In agreement with the polysomal profiling studies, protein synthesis was massively reduced in response to arsenate treatment in both, the control as well as SGD populations.

In summary, the analyses indicated that the combined knockdown of TIA1, TIAR and G3BP1 (SGD) substantially impaired the formation of visible SGs without affecting the upregulation of eIF2α phosphorylation or the stress-induced block of bulk mRNA translation.

### SG assembly: dynamic recruitment of mRNPs versus rigid prion-like scaffolds

Overall our studies were in agreement with the current view that TIA proteins and G3BP1 are essential for the formation of SGs. However, the depletion of TIA proteins or G3BP1 only delayed the formation of SGs, whereas their combined knockdown severely diminished SG formation in a sustained manner. This suggested that either these proteins serve redundant functions as prion-like scaffolding or glue-like factors in the assembly of SGs or that they act redundantly in dynamically recruiting mRNPs to SGs. In line with the prion-like scaffolding scenario one would expect the three factors have an average SG half-life longer than observed for other SG-localized RBPs for which no obvious role in SG assembly has been reported, e.g. IGF2BP1 (16). Moreover, it is tempting to speculate that a prion-like scaffolding factor shows some sort of an immobile fraction in SGs, whereas this should be less pronounced for the transiently stored ‘cargo’, the mRNPs comprising translationally stalled mRNAs and RBPs like IGF2BP1. Aiming to evaluate these aspects, we analyzed the SG dynamics of the prion-like TIA1, TIAR and G3BP1 proteins versus three RBPs (IGF2BP1, YB1 and HUR) that are localized to SGs. As also demonstrated for HUR and YB1 (9), all three analyzed RBPs were dispensable for the assembly of SGs, since SG formation remained unaffected upon the depletion of each protein (IGF2BP1, HUR and YB1) alone as well as the concomitant knockdown of all three RBPs (Supplementary Figure S7A–H).

**Figure 2.**
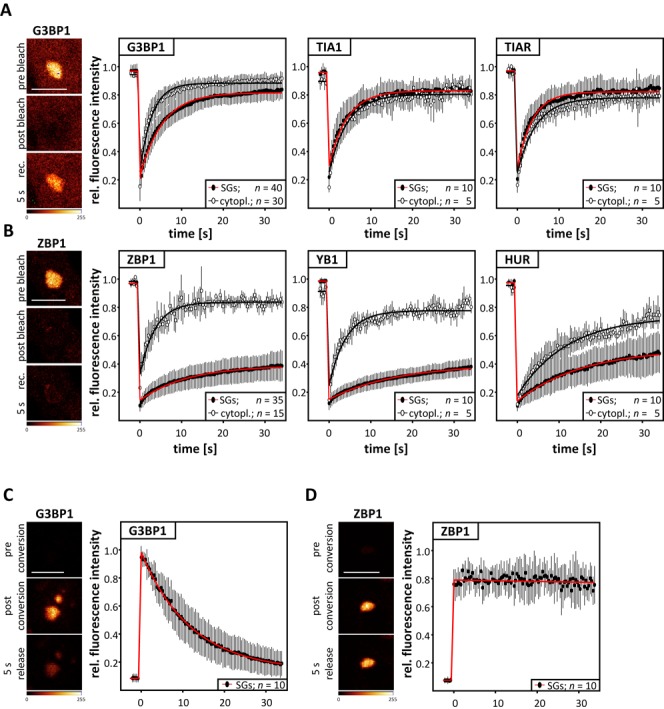
SG dynamics distinguish two classes of SG-associated RBPs. (**A** and **B**) The averaged recovery of GFP-fluorescence for the indicated proteins in SGs (red) or SG-free cytoplasm (black) in U2OS cells stably expressing and/or transiently transfected with the indicated proteins was determined by FRAP. Representative images of fluorescence intensities observed in SGs for GFP-G3BP1 (A) or GFP-ZBP1 (B) at indicated times of FRAP studies are shown in pseudo-colors (glow over/under) in the left panels. (**C** and **D**) The change in SG-localized photo-converted Dendra-fused G3BP1 (C) or ZBP1 (D) was determined by time-lapse microscopy. The averaged change of fluorescence intensities was determined over 30 s after photo-conversion. Representative images of fluorescence intensities observed at indicated time after photo-conversion are shown in left panels. Error bars indicate SD determined for the number of analyses summarized together with kinetic data in Supplementary Figure S8A. Bars, 5 μm.

The dynamics of all proteins were first analyzed by FRAP in cells transiently and/or stably expressing the GFP-tagged fusion protein of interest (Figure 2A and B; Supplementary Figure S8A). Although we aimed at analyzing stably expressed proteins where feasible, we did not observe significant kinetic differences between stably versus transiently expressed proteins (Supplementary Figure S8). Surprisingly, however, TIA1, TIAR as well as G3BP1 showed a short half-live of ∼2–3 s and an insignificant immobile fraction in SGs (Figure 2A, red; Supplementary Figure S8). The latter was consistent with previous studies showing an almost complete fluorescence recovery for TIA1, G3BP, TTP and CPEB (24,29). However, previous studies suggested that the immobile fraction of CPEB in SGs was dependent on whether granules were induced by arsenite or CPEB overexpression (29). In contrast, we did not observe any sort of stressor-dependent differences in G3BP1 dynamics (Supplementary Figure S8A). Notably, GFP-G3BP1 signal recovered fast and almost complete in both, early and small (5–15 min after stress-induction) as well as late and large SGs (30–60 min after stress induction; Supplementary Figure S8B and D). However, it has to be noted that the kinetic models fitted to the FRAP data rely on the assumption that the state of equilibrium has been reached. This severely biases studies at early time points of stress induction and thus the phase of SG initiation. Aiming to compare SG dynamics with the movement of proteins in the cytoplasm, the latter was analyzed by FRAP in the SG-free cytoplasm of stressed cells (Figure 2A, black). Strikingly, the dynamics of TIA1 and TIAR were essentially indistinguishable between SGs and SG-free cytoplasm suggesting that both proteins are rapidly exchanged and remain highly dynamic irrespective of their subcellular localization. Although slightly less mobile in SGs, this was also observed for G3BP1 (Figure 2A; Supplementary Figure S8).

In sharp contrast to the rapid exchange of TIA proteins and G3BP1, the three RBPs dispensable for SG formation (IGF2BP1, YB1 and HUR) showed significantly longer half-lives (∼15–20 s) and a striking immobile fraction (∼60–70%) in SGs (Figure 2B, red, Supplementary Figure S8A). Although less mobile in the cytoplasm when compared to TIA proteins or G3BP1, the analyzed RBPs were substantially more dynamic in the SG-free cytoplasm than in SGs (Figure 2B, black; Supplementary Figure S8A). Notably, the substantially distinct dynamics of TIA proteins and G3BP1 versus other RBPs like IGF2BP1 were observed in early as well as late SGs (Supplementary Figure S8B–D). As for G3BP1, the recovery time of RBPs like IGF2BP1/ZBP1 in early SGs was shorter. This suggests that the accumulation of mRNPs in SGs could be pronounced at early time point of stress induction. This conclusion is of course limited due the fact that an equilibrium has not been reached and thus the conclusiveness of FRAP studies remains limited, as out lined above. Taken together, the presented findings indicated that the prion-like scaffolding TIA proteins and G3BP1 were rapidly turned over in SGs, whereas the RNA- and thus ‘cargo’-associated RBPs were substantially less dynamic. This was largely consistent with previous reports indicating complete fluorescence recovery for G3BP1 or TIA1 but a substantial immobile fraction for the PABP as well as slow and incomplete exchange of CPEB between arsenite-induced SGs and cytoplasm (24,29).

To access SG association more directly, the ‘residence-time’ of one representative of the two classes of SG-localized proteins, G3BP1 versus IGF2BP1, were transiently expressed as Dendra-fusion proteins in U2OS cells (Figure 2C and D). Dendra is a GFP, which is photo-convertible to a RFP by a UV-irradiation or short wavelength lasers (e.g. 405 or 488 nm). This allowed analyzing how rapidly the SG-localized proteins were exported to the cytoplasm when the Dendra-fusion protein had been photo-converted in a single SG (green to red). Consistent with a rapid exchange determined by FRAP, photo-converted Dendra-G3BP1 was cleared from SGs within seconds. In contrast, barely any SG-localized Dendra-IGF2BP1 was lost 30 s after photo-conversion.

In summary, the FRAP and photo-conversion analyses indicated at least two classes of SG-recruited RBPs: (i) proteins essential for the assembly of SGs including G3BP1 and TIA proteins which were rapidly exchanged; (ii) RBPs like IGF2BP1, YB1 or HUR which were dispensable for SG assembly but barely exchanged with the SG-free cytoplasm.

### G3BP1 promotes SG assembly by the RNA-dependent association with RBPs

The dynamics of TIA proteins or G3BP1 suggested that these factors could facilitate the dynamic aggregation of mRNPs in SGs by delivering protein-RNA complexes to these *foci*. Accordingly, the association of these ‘mRNP-movers’ with mRNAs and/or other RBPs like IGF2BP1 should be pronounced during cell stress. Since IGF2BP1 was previously reported to associate with G3BP1 in differentiated P19 neuronal cells (30), we characterized the association of IGF2BP1 and G3BP1 during cellular stress. HEK293 cells were used for these studies due to the high abundance of IGF2BP1 (20).

**Figure 3.**
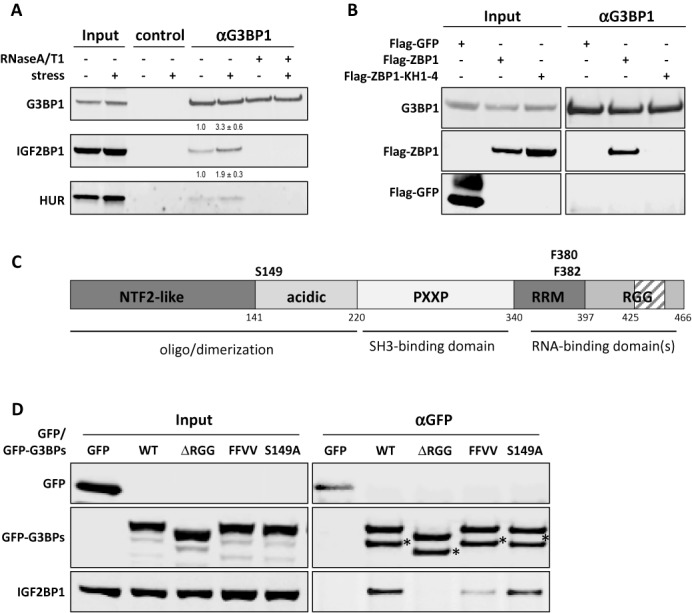
The association of G3BP1 with IGF2BP1 and HUR is enhanced during cellular stress. (**A**) The co-immunopurification of IGF2BP1 or HUR with G3BP1 from arsenate-stressed (+) or non-stressed (−) HEK293 cells was analyzed by western blotting. Where indicated, lysates were supplemented with RNaseA/T1. ProteinG dynabeads served as negative control. Copurification of IGF2BP1 or HUR with G3BP1 was quantified relative to immunopurified G3BP1 amounts by quantitative western blotting. The ratio of IGF2BP1/HUR copurified with G3BP1 was set to one, as indicated above by numbers above lanes. Standard deviation of copurification was determined from three independent experiments. (**B**) The co-immunopurification of endogenous G3BP1 with indicated proteins transiently expressed in HEK293 cells was analyzed by western blotting. Note that RNA binding of ZBP1-KH1-4 is substantially impaired by point mutation in all four KH domains (17). Flag-GFP served as negative control. (**C**) Schematic of G3BP1 domains, putative functions of the indicated domains and relative position of domains indicated by the numbering of residues. (**D**) Co-immunopurification of endogenous IGF2BP1 with indicated stably expressed GFP or GFP-fused G3BP mutant proteins. G3BP proteins analyzed: *WT*, wild type G3BP1; Δ*RGG*, G3BP1 lacking amino acids 425–466; *FFVV*, full-length G3BP1 with F-V conversion at residues 380 and 382; *S149A*, full-length G3BP1 with S-A conversion at residue 149. Western blotting for indicated proteins is shown for the input or co-immunopurified (α-GFP) protein fraction. HEK293 cells stably expressing GFP served as negative controls. * indicates degradation product.

Consistent with previous studies, IGF2BP1 copurified with G3BP1 in a RNA-dependent manner from both, stressed as well as non-stressed HEK293 cells (Figure 3A). In stressed cells, the amount of IGF2BP1 copurified with G3BP1 in a RNA-dependent manner was modestly but reproducibly increased, the amount of IGF2BP1 copurified with G3BP1 in a RNA-dependent manner was modestly but reproducibly increased. This was also observed for the association of G3BP1 with HUR. In agreement, G3BP1 also associated with stably expressed Flag-tagged ZBP1, the chicken ortholog of human IGF2BP1 (Figure 3B), which was previously shown to recover or mimic IGF2BP1-dependent phenotypes (17). Binding was abolished when all four KH-domains were inactivated by point mutations (ZBP1-KH1-4; (17)) in the GXXG loop indicating that IGF2BP1/ZBP1 and G3BP1 associate indirectly via (m)RNA. Notably, we previously demonstrated that the ZBP1-KH1-4 mutant does not localize to SGs suggesting that the SG recruitment of IGF2BP1/ZBP1 essentially relies on RNA binding (17).

The role of G3BP1 in modulating SG formation was suggested to rely on the dephosphorylation of the protein at S149 as well as RNA binding (7). Accordingly, we investigated whether the association of IGF2BP1 and G3BP1 is modulated via the acidic domain comprising S149, involves the RRM and/or the C-terminal RGG domain (Figure 3C). To this end, GFP-tagged wild-type and mutant G3BP1 proteins were probed for association with IGF2BP1 by co-immunoprecipitation (Figure 3D). G3BP1 and the non-phosphorylatable G3BP1-S149A copurified with IGF2BP1 from transiently transfected HEK293 cells. This was also observed for the S149E mutant protein (data not shown). The copurification of the RRM mutant protein (FFVV: F380,382V) or the RGG-lacking mutant (ΔRGG) was either severely diminished or abolished, respectively. These findings supported the view that the association of G3BP1 with IGF2BP1 is facilitated indirectly via (m)RNA but does not involve the modification of S149 in G3BP1.

**Figure 4.**
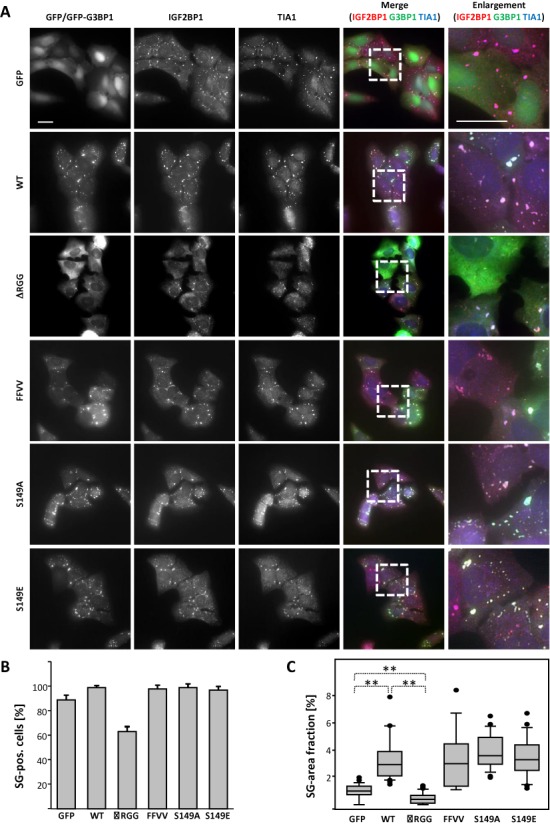
The forced expression of G3BP1 protein mutants modulates SG formation. (**A**) The formation of SGs was monitored in arsenate stressed (1 h) U2OS cells stably expressing GFP or indicated GFP-fused G3BP1 mutant proteins using immunostaining of SG-localized IGF2BP1 and TIA1. Enlargements of boxed regions depicted in the merged images are shown in the right panel. Bars, 25 μm. (**B** and **C**) The number of SG-positive cells (B) as well as the SG-area fraction (C) was determined as described in Figure 1B and C. Error bars indicate SD of at least three independent analyses including at least 30 cells per condition. Statistical significance was determined by Student's *t*-test: **P < 0.005.

G3BPs are considered to act as prion-like nucleators of SGs by enhancing the formation of these foci via their oligomerization (7,28,31). The latter is controlled by the modification of S149, as previously proposed (7). In contrast to the view that the prion-like aggregation of G3BPs and/or TIA proteins provides a rigid and largely non-dynamic scaffold for SG assembly, our analyses of G3BP1 protein dynamics and stress-dependent protein association suggested that the protein promotes SG formation by the dynamic and RNA-dependent recruitment of protein-RNA complexes. To investigate this in further detail, the G3BP1 wild-type and mutant proteins were transiently and stably expressed in U2OS cells. The formation of SGs and recruitment of proteins was monitored by indirect immunostaining of TIA1 and IGF2BP1 (Figure 4A; only shown for stably expressing cells). Consistent with previous reports, transiently expressed G3BP1 induced IGF2BP1- and TIA1- positive SGs in ∼40% of U2OS cells (Supplementary Figure S9A). Although the induction of SGs appeared less pronounced for G3BP1-S149E, both transiently expressed S149 mutant proteins induced the assembly of SGs. Strikingly, however, all G3BP1 mutants with changes in the RNA-binding domains failed to induce SGs when transiently expressed. Although all stably expressed G3BP1 proteins were recruited to SGs (Figure 4A), they failed to induce SG formation in the absence of stress (Supplementary Figure S9A). In agreement, the stress-induced upregulation of eIF2α phosphorylation remained largely unaffected by the stable expression of all proteins (Supplementary Figure S9B). However, compared to GFP-expressing controls, the number of SG-positive cells and more prominently the SG-area fraction were increased in cells stably expressing wild-type G3BP1 (Figure 4A–C). In contrast, both these parameters were significantly decreased in cells expressing the ΔRGG mutant, which also failed to associate with IGF2BP1. The number of SG-positive cells as well as the SG-area fraction was enhanced in cells stably expressing the other G3BP1 mutant proteins.

In conclusion, these findings supported the view that G3BP1 promotes SG assembly in a dose and RNA-binding-dependent manner. In contrast to previous reports (7), the proposed phosphorylation-dependent control of G3BP1-aggregation appeared irrelevant for the formation of SGs in the cells analyzed here. This provided further evidence that instead of acting as a rigid prion- or glue-like scaffold, G3BP1 promotes SG formation by the dynamic RNA-dependent recruitment of mRNPs.

### SGs are dispensable for mRNA stabilization during cellular stress

The concomitant knockdown of TIA proteins and G3BP1 allowed the sustained inhibition of SG formation without affecting stress signaling. This provided a *bona fide* protocol for testing the role of SGs in preventing bulk mRNA degradation during cell stress.

**Figure 5.**
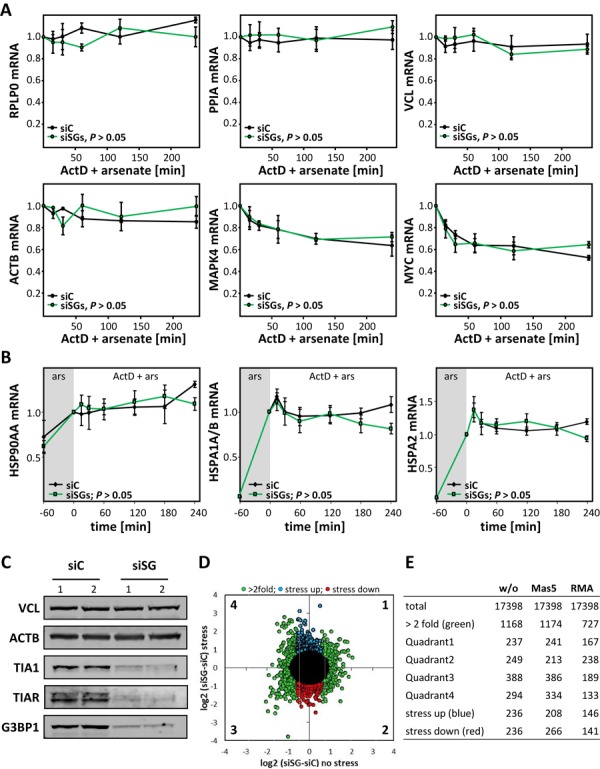
The control of mRNA turnover is independent of SG formation. (**A**) The turnover of indicated mRNAs was analyzed in U2OS cells transfected with indicated siRNAs (siC, control; siSG, siTIA1, siTIAR and siG3BP1) for 72 h. Cells were treated with arsenate and actinomycin D (ActD + arsenate) for indicated times. For RPLP0, PPIA and VCL mRNA abundance was determined relative to untreated samples by the Δ*C_t_* method. For ACTB, MAPK4 and MYC, changes in mRNA levels relative to input (untreated) controls were determined by cross-normalization to RPLP0 using the ΔΔ*C_t_* method. (**B**) The turnover of indicated stress-induced mRNAs was analyzed in U2OS cells transfected as in A. Before monitoring mRNA turnover by ActD addition, cells were pre-stressed by arsenate for 1 h (gray). RNA levels observed after 1 h of arsenate stress were set to one. The decay of mRNAs was analyzed by qRT-PCR using the ΔΔ*C_t_* method and RPLP0 for internal cross-normalization as in A. Error bars indicate SD of at least three independent analyses. Statistical significance was determined by Student's *t*-test. (**C–E**) The abundance of RNAs in stressed (arsenate and ActD for 2 h) and non-stressed U2OS cells transfected as in A was analyzed by comparative microarray analyses. The concomitant knockdown of indicated proteins was confirmed by western blotting with VCL and ACTB serving as internal controls (C). The change of RNA abundance in response to the triple knockdown (siSG, as in A) was determined relative to siC-transfected controls (D). The siSG/siC ratio of transcripts is shown for the average of two independent analyses in stressed and non-stressed U2OS cells. Transcripts with an at least 2-fold distance to the origin in any direction are indicated in green. Transcripts selectively decreased at least 2-fold in the siSG-transfected populations during stress without significantly changed abundance under non-stressed conditions are indicated in red. Transcripts selectively upregulated during stress are indicated in blue. The number of transcripts in the depicted color-coded classes (D) or with an at least 2-fold distance to the origin in the four quadrants (D) was determined without any normalization of array data (E, w/o) or using indicated tools for normalization (E, Mas5 or RMA).

At first, we monitored the turnover of six mRNAs in arsenate-stressed U2OS cells concomitantly transfected with G3BP1-, TIA1- and TIAR-directed siRNAs (SGD; siSGs). The decay of mRNAs was determined upon the block of transcription by actinomycin D (ActD) using qRT-PCR (Figure 5A). Compared to control transfected cells (siC, black), the turnover of three none IGF2BP1-associated transcripts (RPLP0, PPIA and VCL) as well as three IGF2BP1-target mRNAs (ACTB, MAPK4 and MYC) was essentially unchanged by the SGD (siSG, green). Although instable mRNAs like MYC are stabilized during stress, they are still partially degraded. The stress-dependent stabilization of such mRNAs presumably occurs irrespective of SGs, since mRNA decay during cellular stress appeared unchanged when the formation of SGs was impaired by the SGD (Figure 5A). To monitor how the SGD affects the turnover of transcripts induced during the stress response, we analyzed the decay of three mRNAs encoding heat-shock proteins (HSPs), HSP90 (HSP90AA) and HSP70 transcripts (HSPA1A/B; HSPA2), during arsenate-induced cellular stress. For these analyses, cells were stressed by arsenate for 1 h to induce or boost the synthesis of HSP-encoding transcript before blocking transcription by ActD. Like for none stress-induced mRNAs, transcript turnover was monitored by qRT-PCR (Figure 5B). The synthesis of the analyzed HSP-encoding transcripts was enhanced by arsenate confirming the induction of the cellular stress response (Figure 5B; gray). Notably, the induction of mRNA synthesis appeared largely unaffected by the SGD (Figure 5B; compare black to green). Strikingly, HSP transcript levels remained stable over 4 h of ActD treatment in both cell populations, controls (siC, black) versus SGD cells (siSGs, green). However, we cannot exclude that elongated stress conditions might reveal significant differences for HSP-encoding transcripts due to either the inhibition of SG formation or depletion of TIA/G3BP proteins, respectively. Together our data suggested that the stabilization of specific mRNAs could be uncoupled from the formation of SGs during cellular stress. Aiming to monitor bulk mRNA turnover in stressed cells, steady-state mRNA levels in non-stressed and arsenate-treated control versus SGD cells were monitored by comparative microarray analyses (Figure 5C–E; Affymetrix HG133plus2.0). The SGD-induced change in mRNA abundance before stress induction (no stress) or in cells treated with arsenate and ActD for 2 h (stress) was monitored by normalization to siC-transfected controls. These analyses revealed that the SGD failed to induce a significant shift in bulk mRNA levels in stressed or non-stressed cells (Supplementary Figure S10A). This was analyzed in further detail by determining the log2-fold change in transcript abundance induced by the SGD in stressed versus non-stressed cells (Figure 5D and E). Irrespective of the normalization method used, the SGD led to an at least 2-fold up- or down-regulation of 1168 (6.7%) out of 17 398 reliably detected hits (Figure 5D, green). However, the significantly deregulated hits were essentially equally distributed over all four quadrants depicted in the complex dot plot (Figure 5D and E). Moreover, only 236 (1.2%) of all hits were more than 2-fold downregulated in stressed cells due the SGD (Figure 5D, red). However, the same number of hits was upregulated or reduced by the SGD in the absence of stress (Figure 5D, blue). These results remained essentially unchanged by the normalization method or evaluation platform used for analyzing the microarray data suggesting that steady-state bulk mRNA abundance was largely unaffected by the SGD in stressed and non-stressed cells (Figure 5E). To test how the SGD affects the turnover of bulk mRNA, transcript abundance was monitored in cells treated with arsenate and ActD for 30–120 min. As before, mRNA abundance was monitored by microarrays, this time using another type of chip (Illumina; Solexa HumanHT-12). The decay rate of individual transcripts was calculated by the decline of signal intensities determined by microarray studies (Supplementary Figure S10B). For each of the reliably detectable hits (14 338), the ‘slope’ of mRNA degradation in control cells (siC-transfected) was plotted versus the rate of degradation determined in SGD cells. Consistent with barely affected steady-state levels, the decay of bulk mRNA appeared largely unaffected by preventing the formation of SGs. This was also validated by Pearson's correlation analyses of the determined decay rates (Supplementary Figure S10B).

In conclusion, the presented findings provided strong evidence that bulk mRNA turnover remained largely unaffected by preventing the formation of visible SGs by the concomitant knockdown of TIA proteins and G3BP1.

### IGF2BP1-directed mRNA stabilization during cellular stress is independent of SGs

**Figure 6.**
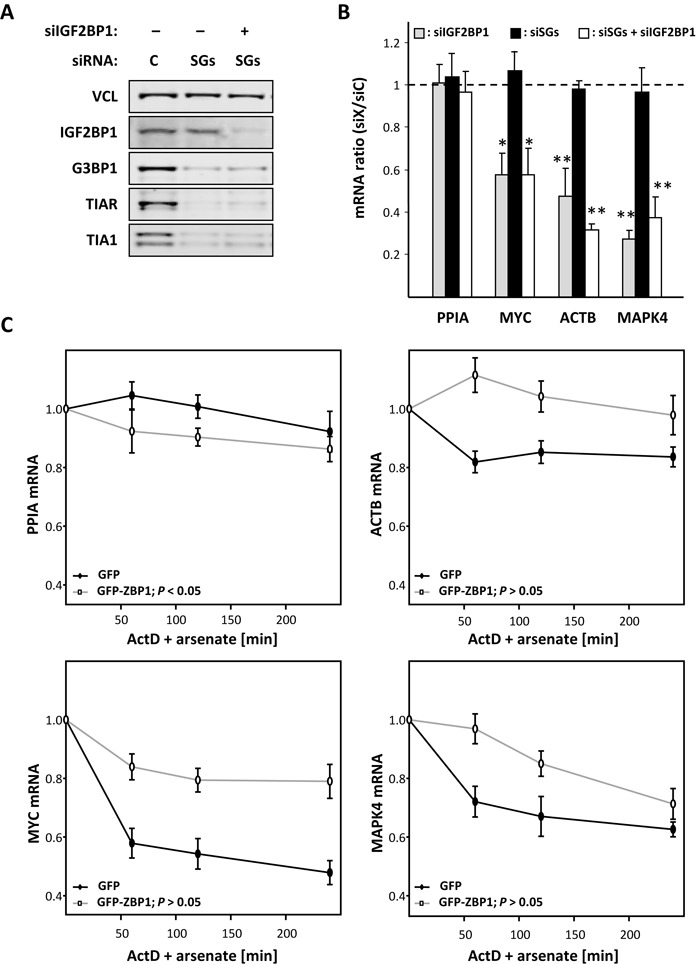
IGF2BP1 stabilizes target mRNAs in a SG-independent manner. (**A**) The knockdown of indicated proteins in U2OS cells transfected with indicated siRNAs or siRNA mixtures (C, control; SG, siTIA1, siTIAR and siG3BP1) for 72 h was analyzed by western blotting. VCL served as loading control. (**B**) The abundance of indicated mRNAs in cells transfected as in (A) and stressed by arsenate for 2 h was determined by qRT-PCR relative to controls (siC) using the ΔΔ*C_t_* method and RPLP0 for internal cross-normalization. (**C**) The turnover of indicated mRNAs was analyzed in U2OS cells stably expressing GFP (black) or GFP-tagged ZBP1 (gray) treated with ActD and arsenate for indicated time. The abundance of indicated mRNAs was analyzed by qRT-PCR relative to untreated samples using the ΔΔ*C_t_* method and RPLP0 for internal cross-normalization. Error bars indicate SD of at least three independent analyses. Statistical significance was determined by Student's *t*-test. *P < 0.05; **P < 0.005.

After having shown that the formation of visible SGs is dispensable for bulk mRNA stabilization during cellular stress, it remained to be addressed if the selective stabilization of IGF2BP1-target mRNAs is maintained when SG formation is impaired. Therefore, we monitored the fate of three IGF2BP1-target transcripts (MYC, ACTB and MAPK4) as well as one none IGF2BP1-associated mRNA (PPIA) upon preventing SG formation in arsenate-stressed cells depleted for IGF2BP1 (Figure 6A and B). Consistent with previous studies (16), target mRNA levels were reduced by the knockdown of IGF2BP1, whereas PPIA transcript abundance remained largely unaffected (Figure 6B, gray). The inhibition of SG formation (SGD) itself had no effect on the abundance of any of the analyzed transcripts supporting the view that preventing SG formation does not affect bulk mRNA turnover (Figure 6B, black). Most strikingly, however, only the IGF2BP1 target mRNAs were decreased in their steady-state levels when the SGD was combined with the knockdown of IGF2BP1 (Figure 6B, white). Compared to the knockdown of IGF2BP1 alone, the decrease in target mRNA abundance was similar to the quadruple knockdown of TIA1, TIAR, G3BP1 and IGF2BP1. This suggested that the target mRNA-specific stabilization by IGF2BP1 is facilitated via IGF2BP1-containing mRNPs but independent of SGs. In line with this conclusion one would expect that increasing the abundance of the stabilizing factor IGF2BP1 promotes the stability of target transcripts during cellular stress due to their increased recruitment into mRNPs. This was tested by monitoring mRNA turnover in stressed U2OS cells stably expressing GFP or GFP-ZBP1 (Figure 6C). The overexpression of GFP-ZBP1 (gray) interfered with the turnover of its target mRNAs MYC, ACTB and MAPK4, as evidenced by significantly elevated mRNA abundance compared to GFP-expressing controls. In contrast, the turnover of none IGF2BP1-associated PPIA mRNA remained largely unaffected by the stable expression of GFP-ZBP1.

In conclusion, our findings indicated that the selective stabilization of target mRNAs by IGF2BP1 was dose-dependent but facilitated in a SG-independent manner.

## DISCUSSION

In this study, we demonstrate that the stabilization of mRNAs during cellular stress can be uncoupled from the aggregation of mRNPs to cytoplasmic SGs visible by fluorescence microscopy. However, RBPs like IGF2BP1 protect their target mRNAs from decay during cellular stress. This mRNA stabilization is facilitated in a dose-dependent manner suggesting that the RBPs recruit target mRNAs to protective mRNPs, as proposed for IGF2BP1 previously (20,32,33). The protective mRNPs apparently form stable RNA-protein complexes, which can transiently assemble into SGs. Consistently, FRAP and photo-conversion studies reveal that RBPs like IGF2BP1 shielding target mRNAs from degradation, presumably by recruiting these into mRNPs, are stably incorporated in SGs. In contrast, RBPs essential for the assembly of SGs but dispensable for bulk mRNA stabilization, for instance, TIA proteins and G3BPs, only transiently associate with SGs. This suggests that they continuously and dynamically recruit mRNPs to SGs instead of forming a rigid and largely non-dynamic protein scaffold for the assembly of SGs.

### SGs are dispensable for the control of bulk mRNA fate in stressed cells

There is accumulating evidence that the assembly of mRNPs in super-structures during cellular stress is largely dispensable for the control of bulk mRNA translation, whereas relatively little is known about the role of SGs in controlling mRNA turnover [reviewed in (13,34)].

A putative role of SGs in controlling protein synthesis is largely based on the observation that key factors modulating mRNA translation accumulate or transiently associate with SGs [reviewed in (2,24)]. Experimental proof supporting roles of SGs in controlling mRNA translation, however, remain sparse. On the contrary, recent findings provide strong evidence that the assembly of mRNPs in SGs is even dispensable for controlling mRNA translation during cellular stress. When SG formation is inhibited by the depletion of factors essential for their assembly, the block of mRNA translation observed during cellular stress is maintained (10,11). Moreover, recent findings indicate that during the recovery from hypothermia the complete resumption of mRNA translation is largely uncoupled from SG disassembly (12). Consistently, we demonstrate that the inhibition of SG formation by the combined depletion of G3BP1 and TIA proteins neither affects the phosphorylation of eIF2α nor interferes with the stress-induced block of bulk mRNA translation.

As observed for the control of mRNA translation, it was proposed that SGs also modulate the stabilization of bulk mRNA during cellular stress [reviewed in (13)]. However, in yeast the depletion of factors essential for the assembly of SGs does not impair bulk mRNA stabilization during cellular stress (35). In cancer-derived cells, IGF2BP1 is essential for stabilizing target mRNAs during cellular stress, as shown here and previously (16). However, although IGF2BPs are recruited to SGs (16,17), their role in mRNA stabilization during cellular stress is maintained when the formation of SGs is impaired. Likewise, we observe that the turnover of stress-induced transcripts, for instance, HSP-encoding mRNAs, as well as bulk mRNA turnover remains essentially unchanged when preventing the formation of SGs. In conclusion, these findings provide strong evidence that the assembly of mRNPs in SGs is dispensable for modulating mRNA turnover in cancer-derived cells. This suggests that cytoplasmic mRNA fate during cellular stress is largely if not exclusively determined by the recruitment of mRNAs into comparatively stable protein-mRNA complexes termed mRNPs. We propose that the ‘caging’ of mRNAs in mRNPs transiently protects associated transcripts from being degraded during cellular stress. In support of this view, the increased abundance of mRNP ‘guards’, for instance, IGF2BP1, enhances the stress-dependent stabilization of mRNAs. Moreover, the formation of comparatively stable ‘mRNA cages’ is supported by FRAP and photo-conversion analyses. These provide strong evidence that ‘protective’ RBPs, for instance, IGF2BP1, are barely turned over in SG-recruited mRNPs. Although these mRNPs are assumed to partially cycle between SGs, the cytoplasm and remaining polysomes, they were suggested to be mainly concentrated in the cytoplasm outside SGs (29). How mRNAs are released from their cages and why mRNPs associate into SGs remains to be addressed. However, it is tempting to speculate that post-translational protein modifications of RBPs provide regulatory triggers allowing the control of mRNA association and mRNP-recruitment to SGs.

### Prion-like scaffolding versus dynamic recruitment of mRNPs in SGs

SG formation is considered to essentially rely on the aggregation of proteins comprising LC, also termed prion-like, domains via which they assemble into larger structures. Similar to germ cell granules, this aggregation is expected to induce a ‘demixing phase transition’ resulting in the formation of hydrogel-like structures with a granular morphology, in this case SGs [reviewed in (4,5)]. In agreement with this view, LC domains have been characterized in various RBPs localized to SGs and for some of these proteins aggregation into hydrogel-like superstructures has been demonstrated *in vitro*, e.g. TIA1 (6). Notably, there is accumulating evidence that protein-(RNA) aggregates observed in some human neurodegenerative diseases result from a disturbed SG homeostasis [reviewed in (5)]. The deregulated subcellular sorting and/or mutations in LC domains were suggested to induce aberrant aggregation of LC domain containing proteins like TIA1 or TDP-43 in nuclear and/or cytoplasmic foci. However, recent findings nuance this view by providing evidence that SG formation essentially relies on excessive, non-polysome associated (m)RNA and consistently is induced by free RNA or single-stranded DNA (9).

If TIA proteins and/or G3BPs would facilitate the assembly of SGs by forming a rigid and barely dynamic prion-like scaffold one would expect a reduced turnover rate of these factors in SGs. At least they should reside in SGs longer than proteins, which are dispensable for the formation of SGs, for instance, IGF2BP1. However, we observe the exact opposite, with TIA proteins and G3BP1 having comparatively short half-lives and an insignificant immobile fraction in SGs. The latter is also supported by previous studies reporting insignificant immobile fractions for TIA proteins and G3BP1 (24). Furthermore, the dynamics of TIA/G3BP proteins in SGs were comparable to those measured in the SG-free cytoplasm. In contrast, RBPs found to be dispensable for SG formation but essential for the selective stabilization of mRNAs during cellular stress, are associated with SGs at 4–5 times longer half-lives and strikingly increased immobile fractions. These observations indicate that instead of forming a rigid glue-like scaffold, TIA proteins and G3BP1 dynamically recruit mRNPs to SGs and thus act as ‘mRNP movers’. In support of this view, we and other labs largely failed to purify SGs, whereas the subcomplexes forming SGs, namely, mRNPs, can be isolated from stressed cells by density centrifugation (data not shown). Moreover, we demonstrate that the amount of IGF2BP1 and HUR copurified with G3BP1 during stress is increased. We therefore conclude that G3BP1 and presumably TIA proteins promote the assembly of SGs by the dynamic recruitment of mRNPs to sites of SG formation instead of acting like rigid scaffolds formed by prion-like aggregation. This dynamic recruitment may involve the transient assembly of TIA proteins and G3BPs via ID protein domains. In accord with a redundant role of TIA proteins and G3BPs, the assembly of SGs is only delayed but not prevented when the factors are depleted separately, whereas it is substantially impaired by their concomitant knockdown.

If the presented findings here also apply to cytoplasmic granules observed in neurodegenerative diseases remains to be investigated. Although the formation of SGs in neurons essentially relies on the same proteins (TIA1, TIAR, G3BP1/2 and TTP), their maturation to pathological structures was proposed to involve secondary RBPs like TDP-43, ATXN2 or FUS (36–38). This suggests the assembly and persistence of such granules is modulated in a distinct manner.

### SGs: connecting protein synthesis to stress-signaling?

In previous studies we suggested that IGF2BPs prevent target mRNA degradation during cell stress by recruiting target transcripts to SGs (16). Here, we demonstrate that the stabilization of mRNAs during cellular stress can be uncoupled from the assembly of mRNPs in SGs. Moreover, we propose that the formation of SGs requires the dynamic recruitment of cytoplasmic mRNPs. Notably, these findings do not contradict the suggested role of SGs in modulating cellular signaling, for instance, by sequestering protein-kinases, which in the case of mTOR signaling can antagonize signaling pathways during cellular stress [reviewed in (4,5)]. Moreover, our findings support the hypothesis that SGs play a minor role in modulating mRNA fate during cellular stress. The latter presumably is largely and sufficiently facilitated by mRNPs, as supported by here presented and previous analyses [reviewed in (4,5,13)]. In conclusion, this suggests that SGs, although dispensable for controlling mRNA translation and/or turnover, orchestrate stress signaling and the crosstalk of stalled bulk protein synthesis with protein unfolding during cellular stress. This is supported by the finding that a variety of signaling components, e.g. the mTORC1 complex, RACK1 or the serine/threonine kinase FAST, were recruited to SGs (24,39,40). The latter was shown to have a significant immobile fraction in SGs suggesting a substantial retention time for this kinase in SGs (24). This sequestering of signaling components was, for instance, proposed to enhance cell viability by modestly promoting autophagy via the inhibition of mTOR signaling (41). Furthermore, SGs could provide a dynamic platform for stress-dependent protein modifications by localizing and concentrating signaling molecules of distinct parts of the cell. The mechanisms and consequences of SG-directed regulation of stress signaling remain to be addressed in further detail. Moreover, future studies have to investigate whether protein-RNA aggregates observed in neurodegenerative diseases serve roles in controlling mRNA fate or rather act as signaling modulators as proposed for SGs.

## SUPPLEMENTARY DATA

Supplementary Data are available at NAR Online.

SUPPLEMENTARY DATA
