# Supplementary material for: Stress granules are dispensable for mRNA stabilization during cellular stress
Source: Nucleic Acids Res. 2014 Dec 8;43(4):e26. doi: 10.1093/nar/gku1275 (PMC4344486; doi:10.1093/nar/gku1275)
Supplement: SUPPLEMENTARY DATA [file supp_43_4_e26__index.html]

Stress granules are dispensable for mRNA stabilization during cellular stress — SUPPLEMENTARY DATA 

# Stress granules are dispensable for mRNA stabilization during cellular stress

## SUPPLEMENTARY DATA

**Files in this Data Supplement:**

- SUPPLEMENTARY DATA
- SUPPLEMENTARY DATA
