# Supplementary material for: Stress granules are dispensable for mRNA stabilization during cellular stress
Source: Nucleic Acids Res. 2014 Dec 8;43(4):e26. doi: 10.1093/nar/gku1275 (PMC4344486; doi:10.1093/nar/gku1275)
Supplement: SUPPLEMENTARY DATA [file supp_gku1275_nar-01907-y-2014-File008.pdf]

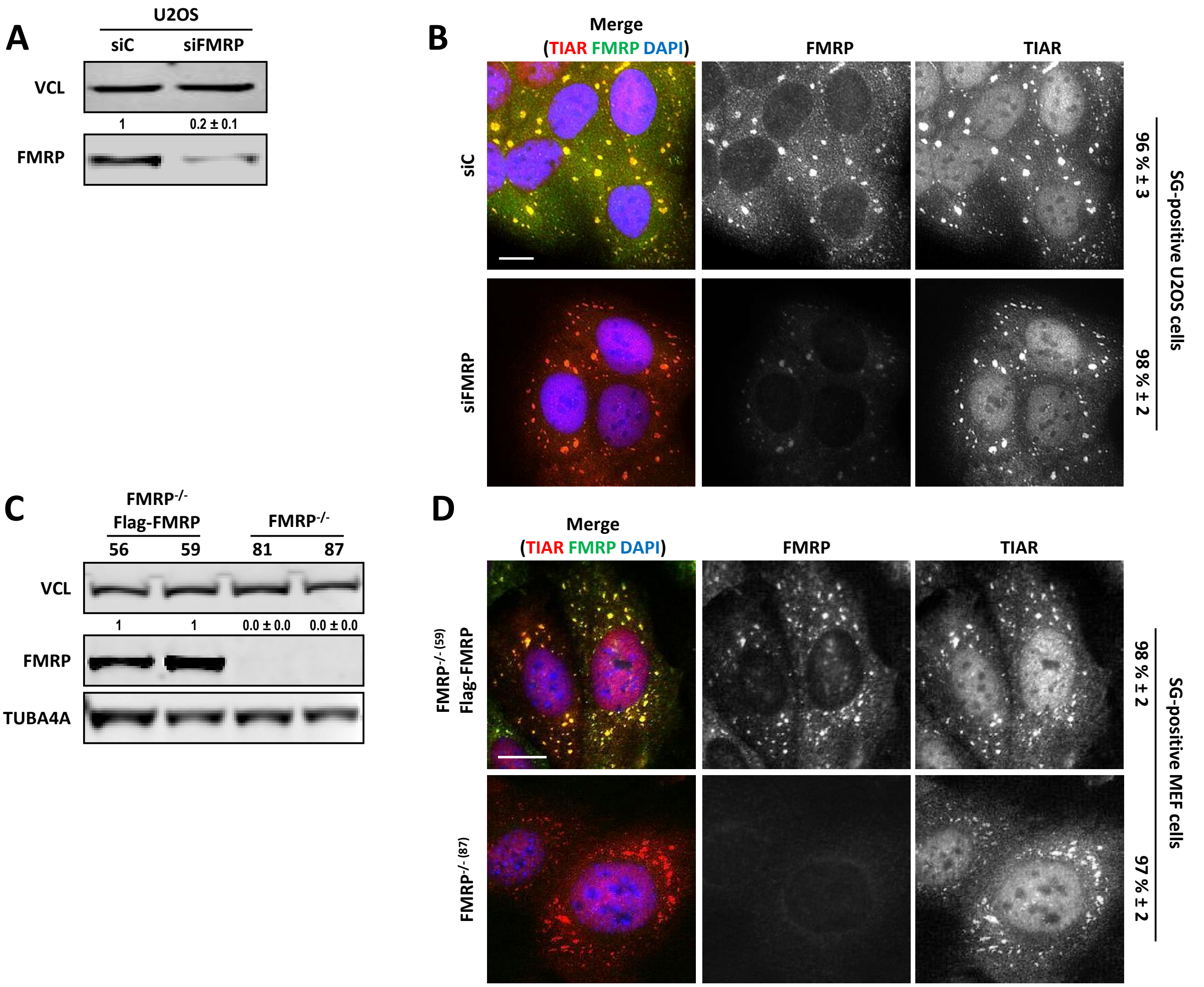

Bley et al., Figure S1

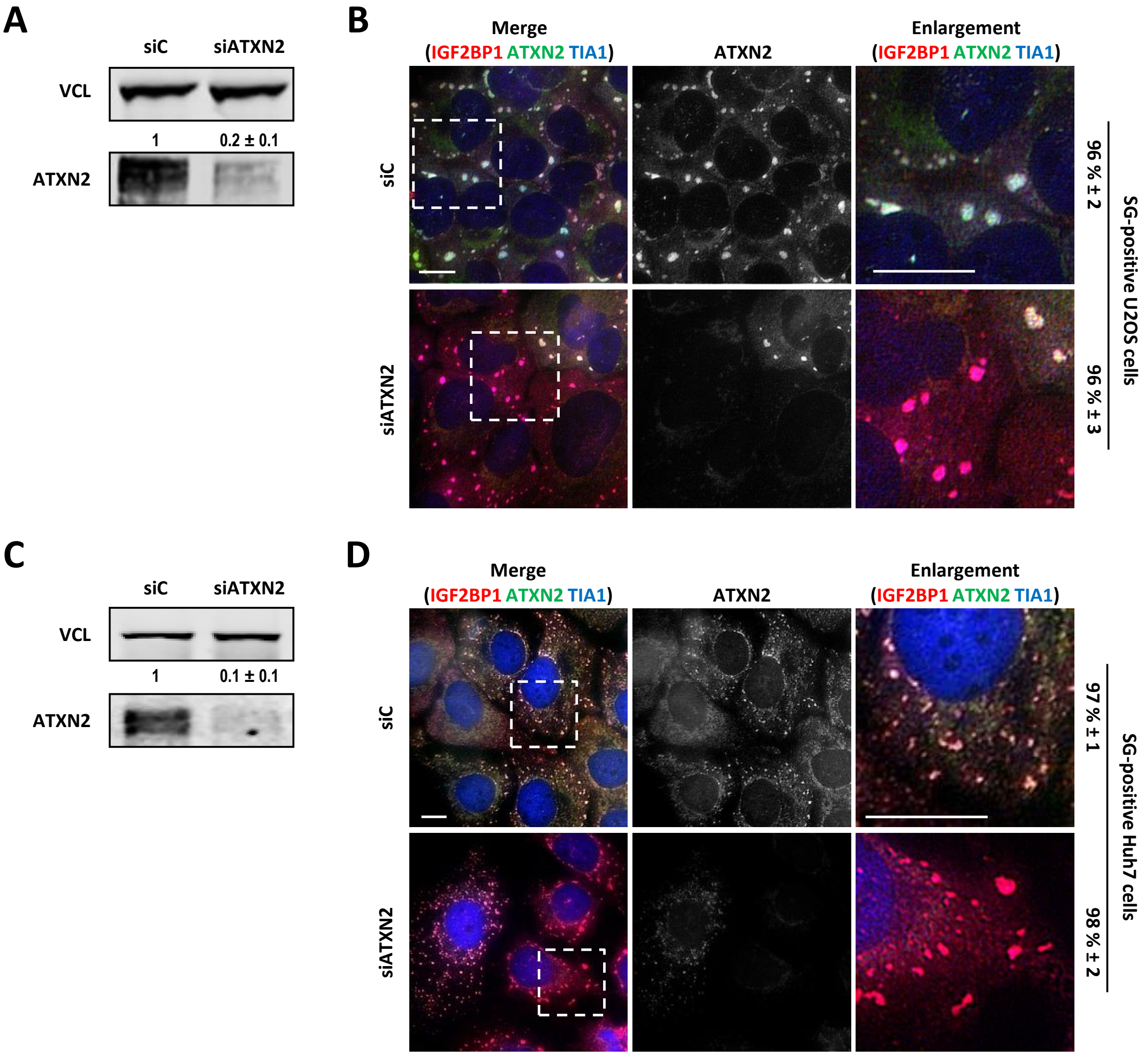

Bley et al., Figure S2

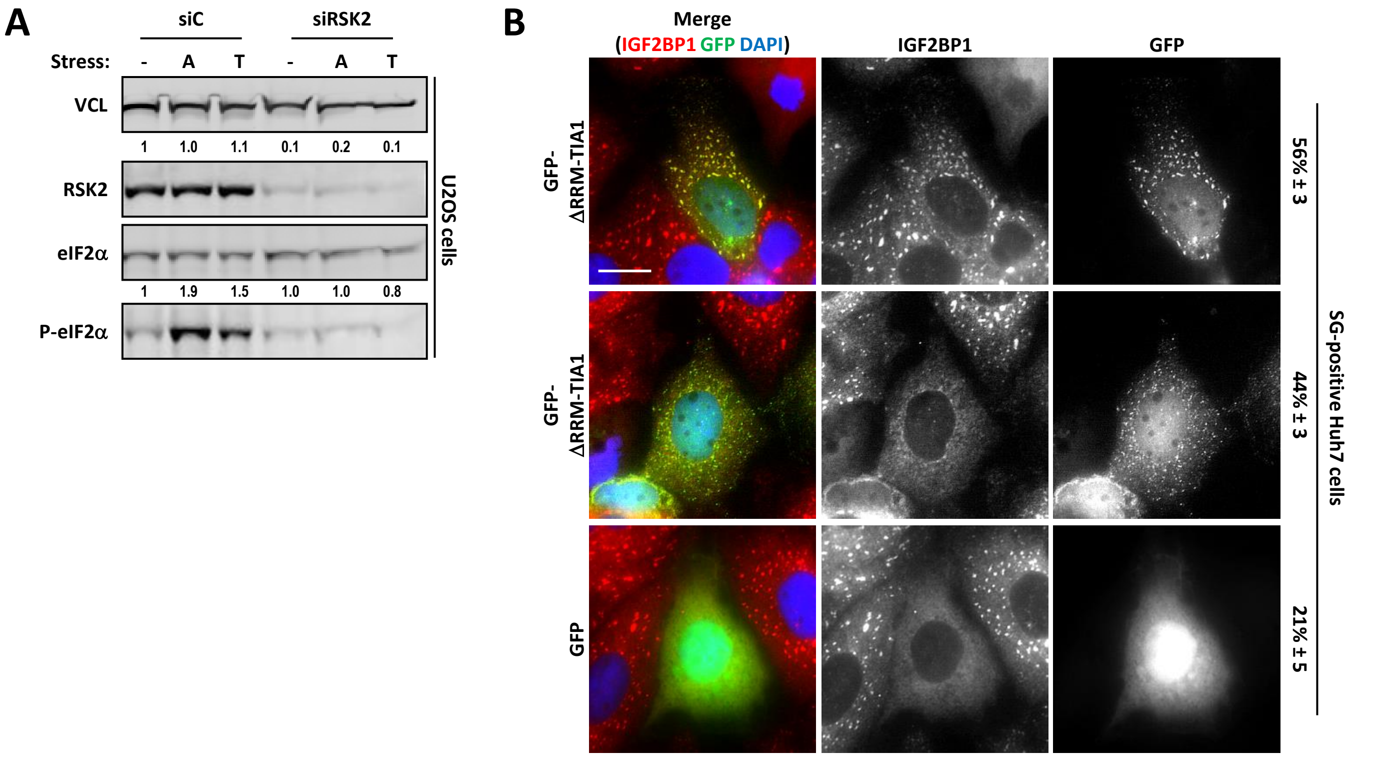

Bley et al., Figure S3

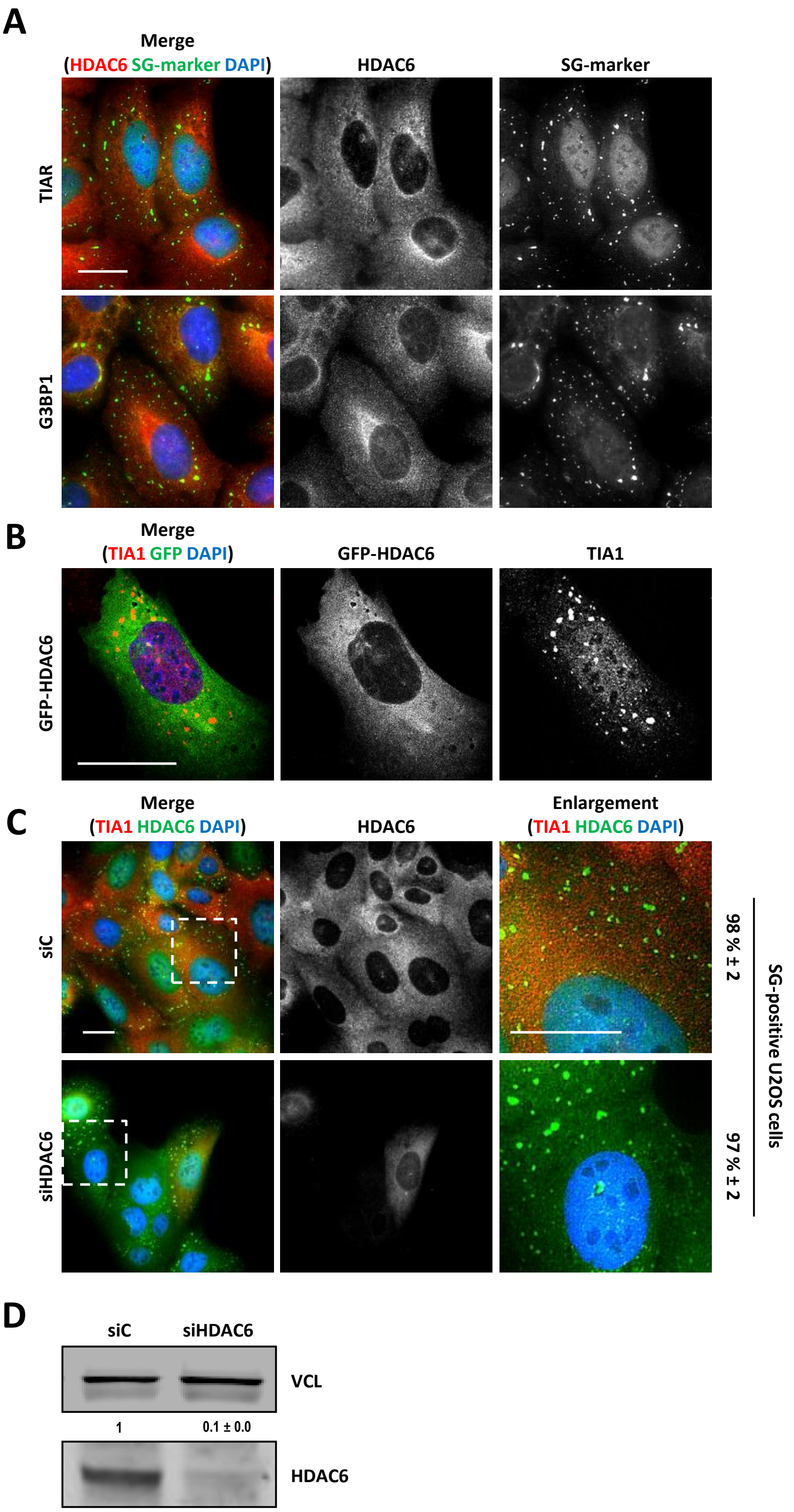

Bley et al., Figure S4

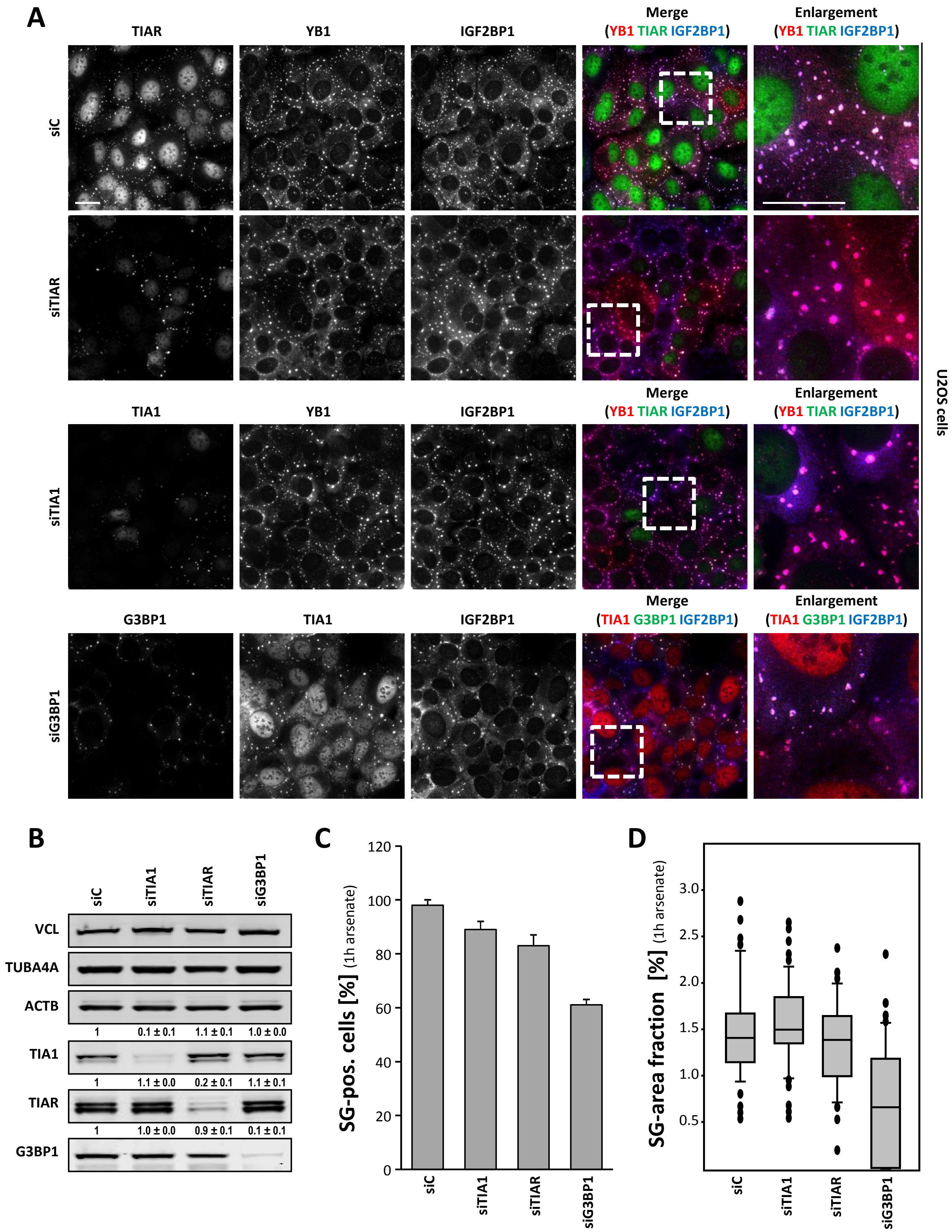

Bley et al., Figure S5

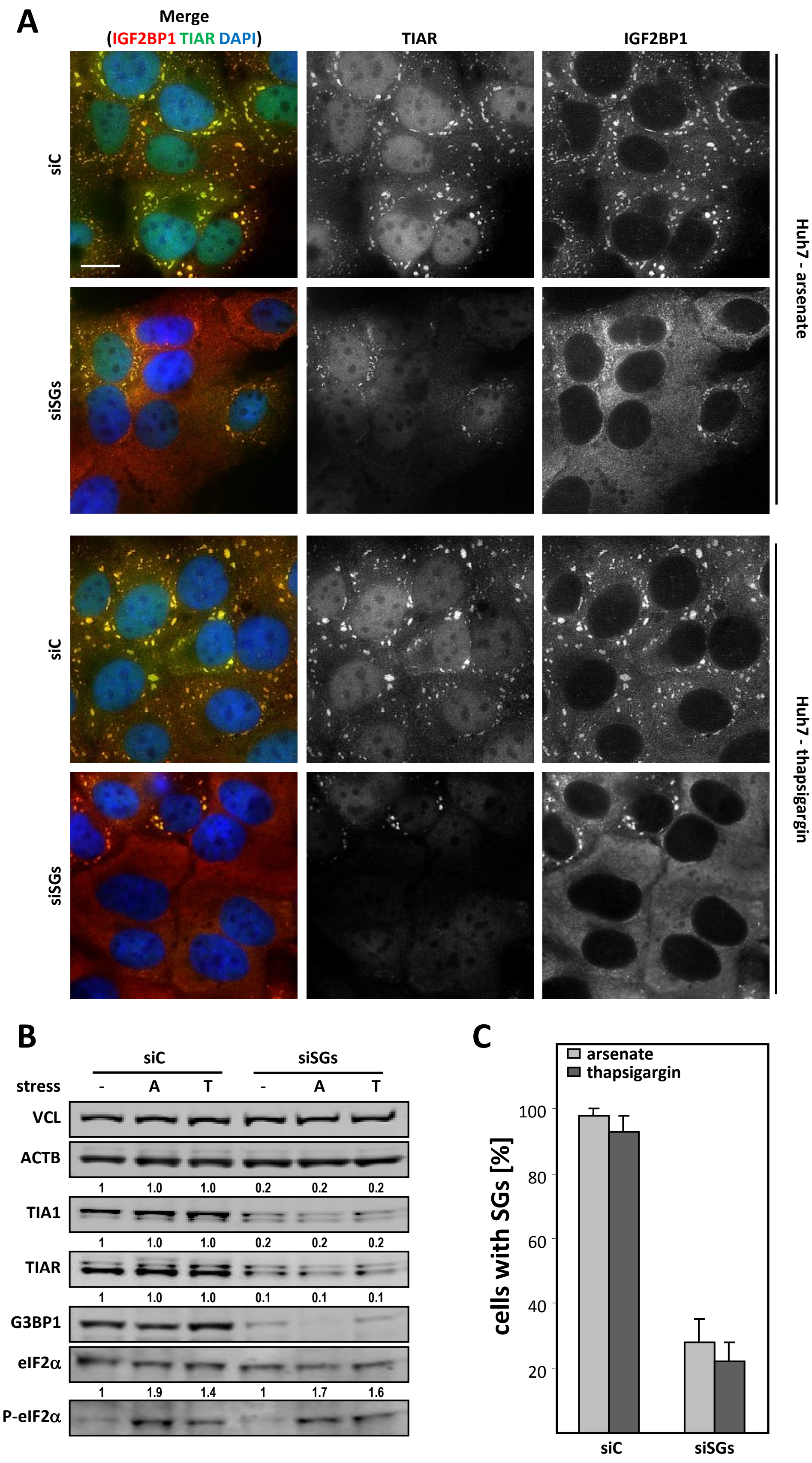

Bley et al., Figure S6

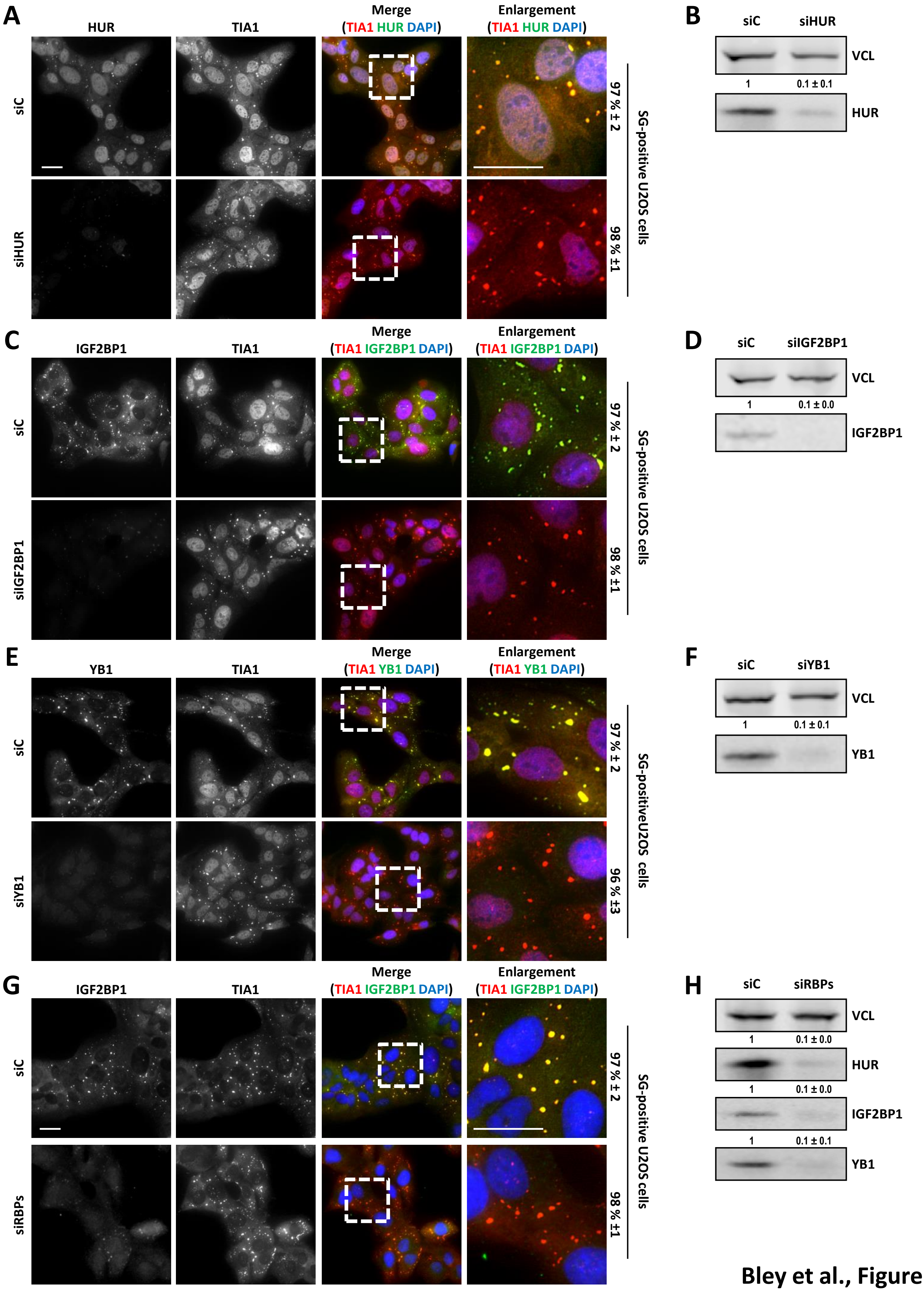

Bley et al., Figure S7

A

protein	conditions	$t_{1/2}$ [sec] SGs	$t_{1/2}$ [sec] cytoplasm	immobile fraction [%] SGs	immobile fraction [%] cytoplasm	experiments SGs	experiments cytoplasm
G3BP1	OE-induced	6,6	1,1	36,2	15,2	10	10
	transient/arsenate	3,4		30,6		10	
	stable/arsenate	3,5	1,8	21,7	15,8	30	20
	all	3,5	1,6	23,7	15,7	50	30
TIA1	transient/arsenate	2,5	2,8	24,4	26,2	10	5
TIAR	transient/arsenate	2,2	3,0	24,9	29,4	10	5
ZBP1	transient/arsenate	13,6	1,9	71,4	25,8	10	5
	stable/arsenate	15,8	2,0	72,5	24,2	25	10
	all	14,8	2,0	72,3	24,6	35	15
IGF2BP1	transient/arsenate	19,5	5,8	71,7	20,9	10	5
HUR	transient/arsenate	17,7	9,3	57,0	31,9	10	5
YB1	stable/arsenate	20,7	2,7	70,9	29,7	10	5

B

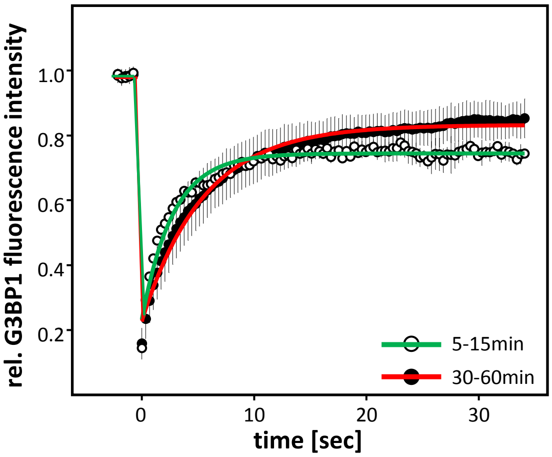

C

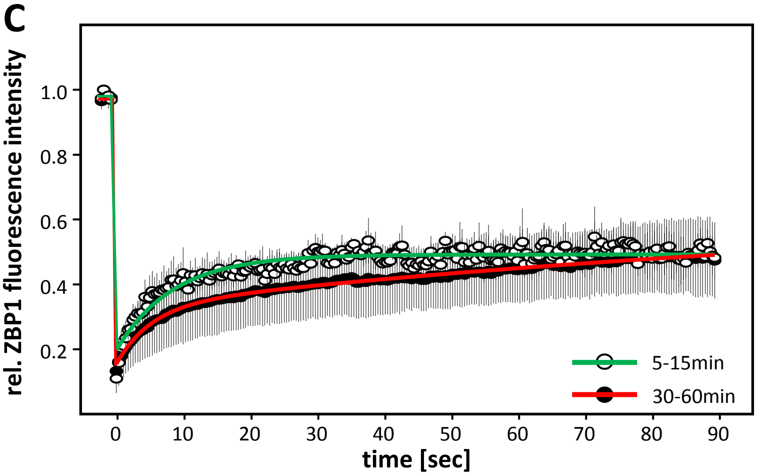

D

**protein**	**arsenate**	**$t_{1/2\ in}$ [sec]**	**IF [%]**
G3BP1	5-15min	1,3	33,3
	30-60min	1,8	21,7
ZBP1	5-15min	7,6	63,2
	30-60min	13,8	66,9

Bley et al., Figure S8

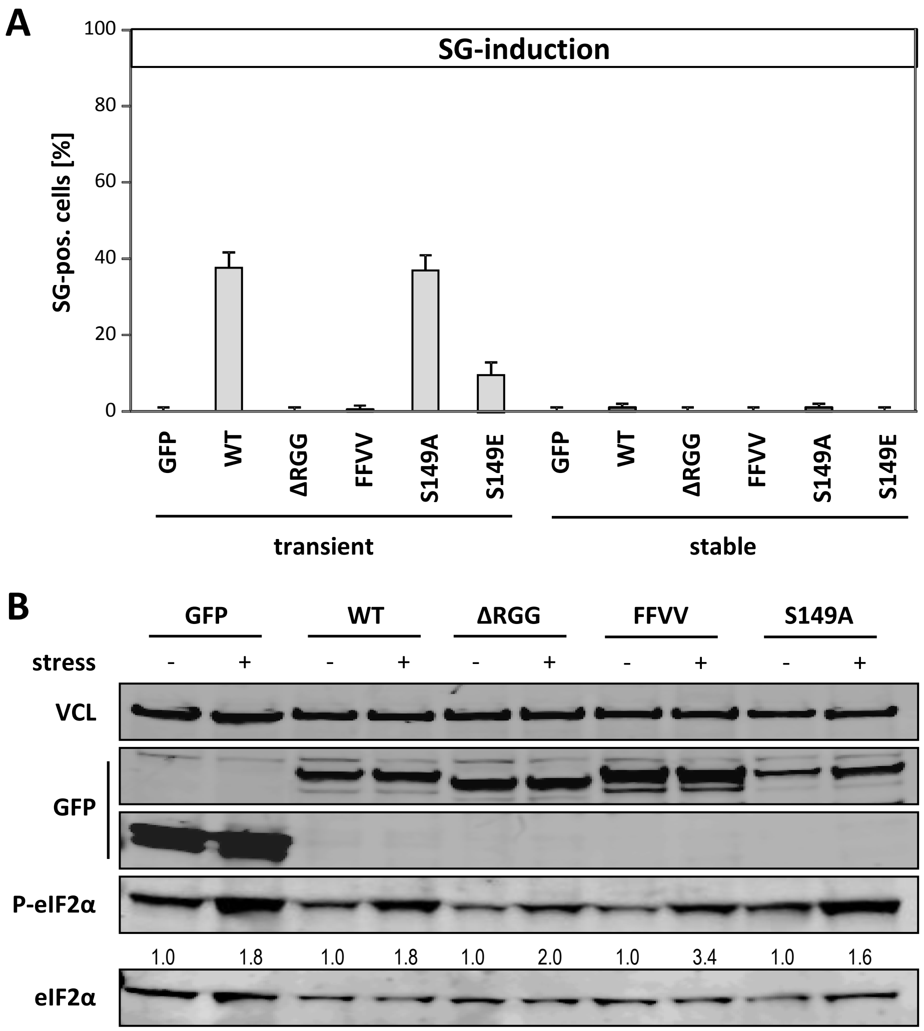

Bley et al., Figure S9

A

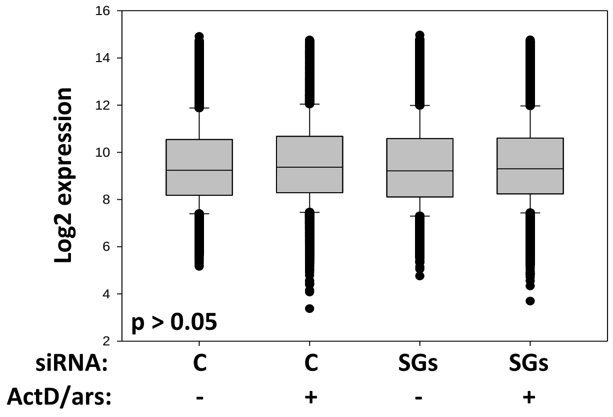

B

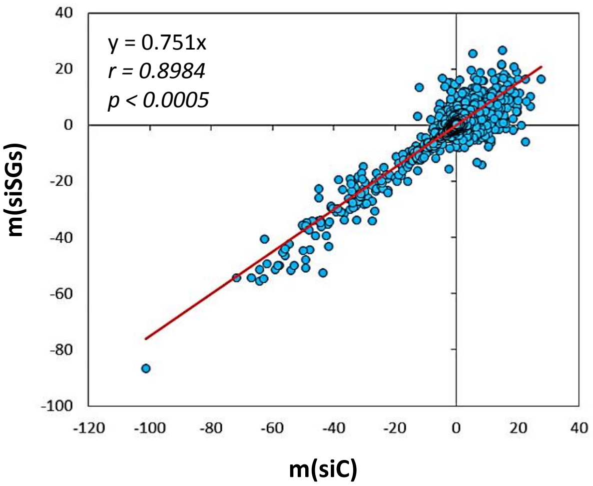

Bley et al., Figure S10

Table S1: siRNAs, plasmids and oligonucleotides

siRNAs	***sequence***			
siC (C.el.mir239b)	uuguacuacacaaaaguacug			
siIGF2BP1	ugaauggccaccaguugga			
siYB1	gcgaagguucccaccuuacua			
siHUR	ggacaaaaucuuacagguu			
siTIA1	auucgagucuuuccagaua			
siTIA1 (2)	caggaaagucuaagggaua			
siTIAR	ugacagaaguccuuauacu			
siTIAR (2)	gaaaggaggucaaaguaaa			
siG3BP1	aaagccugagccaguauua			
siG3BP1 (2)	cauuaacaguggugggaaa			
siRSK2	ccaugaagguauugaaga			
siHDAC6	gguaaagaagaaaggcaaa			
siATXN2	uaugaggaugguucauaua			
plasmids	***vector***	***sense***	***antisense***	***cloning***
GFP-DRRM-TIA1	pEGFP C2	aagaattcatgcgtcagactttttcacc	ctcgagtcactgggtttcataccctgc	EcoRI/XhoI in SalI
GFP-HDAC6	pEGFP C2	aaaga tct atgacctcaaccggccaggattccacc	aacccgggttttcattttctctgtggcccgg	BglII/Xma
		aacccgggagctgcaccgtgagagttccaac	aatctagattagtgtgggtggggcatatcctcccc	Xma/XbaI
GFP-G3BP1	described in Stohr et al. 2006	gggaattcatggtgatggagaagcctagtc	gggtcgacttactgccgtggcgcaagcccccttc	EcoRI/SalI
GFP-G3BP1	pLVX-puro-GFP	gcacgaattcatggtgatggagaagcctagtcccctgctgg	tggcgtcgactcactgccgtggcgcaagccccccttcccac	EcoRI/SalI
GFP-G3BP1-DRGG	pLVX-puro-GFP	gcacgaattcatggtgatggagaagcctagtcccctgctgg	ggcgtcgactcaatctcgtcggtcgccttccctggcagctcg	EcoRI/SalI
GFP-G3BP1-FFVV	pLVX-puro-GFP	ggcgtcgactcaatctcgtcggtcgccttccctggcagctcg	tcagaatcatcaaacacaacaacaccaacattgggtaatttcccac	mutagenesis
GFP-G3BP1-S149A	pLVX-puro-GFP	gagcctcaggaggaggctgaagaagaagtagagg	cctctacttcttcttcagcctcctcctgaggctc	mutagenesis
GFP-G3BP1-S149E	pLVX-puro-GFP	gagcctcaggaggaggaagaagaagaagtagagg	cctctacttcttcttcttcctcctcctgaggctc	mutagenesis
shATXN2	pSuper			
GFP-YB1	pEGFP C2, Clontech			subcloned from pcDNA3.1 via EcoRI/XhoI in SalI
GFP-HUR	pEGFP C2, Clontech			subcloned from pcDNA3.1 via EcoRI/XhoI in SalI
GFP-ZBP1	described in Stohr et al. 2006			
YFP-TIA1	kind gift from R.H. Singer, Albert-Einstein College of Medicine, NYC			
GFP-TIAR	pEGFP C2, Clontech	gggaattcatgatggaagacgacgggcagc	ggctcgagtcactgtgtttggtaacttgccatac	EcoRI/XhoI in SalI
Dendra-ZBP1	pDendra2-C, Clontech, modified MCS			subcloned from pcDNA3.1 via EcoRI/XhoI
Dendra-G3BP1	pDendra2-C, Clontech, modified MCS			subcloned from pcDNA3.1 via EcoRI/XhoI in SalI
qRT-PCR	***sense***	***Antisense***		
HSP90AA	ggtcctgtgcggtcacttag	aaaggcgaacgtctcaacc		
HSPAA/B1	caagatcaccatcaccaacg	tcgtcctccgctttgtactt		
HSPA2	caagatcaccatcaccaacg	cgtcctccgctttgtacttc		
MAPK4	tgctcaagattggggatttc	gatgagttgcatctgctcca		
MYC	agcgactctgaggaggaac	cgtagttgtgctgatgtgtg		
ACTB	agaaaatctggcaccacacc	agaggcgtacagggatagca		
PPIA	gtcaaccccaccgtgttctt	ctgctgtctttgggaccttgt		
RPLP0	ggcgacctggaagtccaact	ccatcagcaccacagccttc		
VCL	ttacagtggcagaggtggtg	tcacggtgttcatcgagttc		

Bley et al., Table S1

Table S2: antibodies

antibodies	***Company***	***Clone number***
anti-IGF2BP1	Stohr et al., 2012	mouse monoclonal
anti-IGF2BP1	Huttelmaier et al., 2005	rabbit polyclonal
anti-YB1	Santa Cruz	59-Q
anti-HUR	Santa Cruz	sc-5261
anti-TIAR	BD Transductions	610352
anti-TIA1	Santa Cruz	N-17
anti-G3BP1	Santa Cruz	H-10
anti-eIF2a	Cell Signaling	9722
anti-phospho-eIF2a (S51)	Cell Signaling	L57A5
anti-RSK2	Cell Signaling	9340
anti-FMRP	Abcam	ab27455
anti-HDAC6	Santa Cruz	H-300
anti-ATXN2	BD Transductions	611378
anti-GFP	Santa Cruz	B-2

Bley et al., Table S2
