# Supplementary material for: Stress granules are dispensable for mRNA stabilization during cellular stress
Source: Nucleic Acids Res. 2014 Dec 8;43(4):e26. doi: 10.1093/nar/gku1275 (PMC4344486; doi:10.1093/nar/gku1275)
Supplement: SUPPLEMENTARY DATA [file supp_gku1275_nar-01907-y-2014-File009.pdf]

SUPPLEMENTARY FIGURE LEGENDS

Figure S1. FMRP is not essential for SG-formation. **(A, B)** U2OS cells were transiently transfected with indicated siRNAs for 72h. FMRP knockdown efficiencies were determined by Western blotting relative to the control population (siC) and VCL serving as loading control (A). The formation of SGs upon arsenate treatment (2.5 mM for 1h) was analyzed by immunofluorescence staining with indicated antibodies (B). The number of SG-positive cells was determined by counting of at least 50 cells from three independent experiments. **(C)** The expression of FMRP in immortalized FMRP (-/-) MEFs stably transfected with Flag-FMRP (56 & 59) or control (81 & 87) vector, as previously described (1), was analyzed by Western blotting as in (A). **(D)** The formation of stress granules by arsenate (2.5 mM for 1h) was analyzed by immunostaining with indicated antibodies and quantified as in (B). Note that the loss of FMRP does neither prevent the formation of SGs in U2OS nor FMRP (-/-) MEFs (data for clones 56 and 81 are not shown). Standard deviation was determined from three independent experiments. Bars 25 µm.

Figure S2. ATXN2 knockdown is insufficient to prevent SG-formation in U2OS and Huh7 cell. **(A-D)** U2OS (A, B) or Huh7 (C, D) cells transfected with indicated siRNAs for 72h were analyzed by Western blotting with indicated antibodies (A, C). Knockdown efficiencies for ATXN2 depletions depicted as numbers above panels were quantified relative to the control (siC). VCL served as loading control. The formation of stress granules was analyzed as in (Figure S1) using indicated antibodies for immunostaining (B, D). Note that the depletion of ATXN2 does not interfere with SG-assembly in both cell lines. Standard deviation was assessed from three independent analyses. Bars 25 µm.

Figure S3. Depletion of RSK2 or overexpression of ΔRRM-TIA1 are inappropriate to inhibit SG formation. (**A**) U2OS cells transfected with indicated siRNAs for 72h and treated with arsenate (A: 2.5mM for 1h) or thapsigargin (T: 1µM for 1h) were analyzed by Western blotting with indicated antibodies. Note that the knockdown of RSK2 impairs the stress-induced phosphorylation of eIF2α and consequently interferes with the assembly of stress granules (data not shown). (**B**) Huh7 cells transfected (48h) with the indicated GFP-tagged TIA1 mutant (ΔRRM) lacking two RRM domains or GFP alone were stressed by arsenate as in (A) before immunostaining with indicated antibodies. In contrast to previous reports (2), the formation of SGs was only modestly impaired by the overexpression of the TIA1-mutant. In approximately 44% of transfected cells, substantially fewer SGs were formed and granules appeared significantly smaller with barely any IGF2BP1. Most notably, however, SG-formation was substantially impaired in 20% of GFP-only transfected cells. Errors indicate s.d. of three independent analyses. Bars 25 µm.

Figure S4. HDAC6 is dispensable for SG-formation. (**A, B**) The localization of endogenous (A) or GFP-tagged (B) HDAC6 in U2OS cells stressed by arsenate (2.5mM, 1h) was analyzed by immunostaining with indicated antibodies. Note that endogenous as well as GFP-tagged HDAC6 are not recruited to arsenate-induced SGs. (**C, D**) U2OS cells transfected with HDAC6-directed (siHDAC6) or control (siC) siRNAs for 72h were treated as in (A) before immunostaining (C; number of SG-positive cells indicated) or Western blotting (D). Knockdown efficiencies were determined by Western blotting relative to the control population (siC) and VCL serving as loading control. Note that in contrast to previous reports (3), HDAC6 is neither recruited to stress granules nor does its knockdown prevent SG-formation in U2OS cells. Errors indicate s.d. of three independent analyses. Bars 25 µm.

Figure S5. The depletion of TIA proteins or G3BP1 alone barely impairs the formation of SGs. (**A-D**) U2OS cells were transfected with indicated siRNAs for 72h and stressed by arsenate (2.5µM) for 1h. SG-assembly was monitored by indirect immunostaining of indicated proteins (A). Enlargements of the boxed regions in the merged images are shown in the right panel. The knockdown of indicated proteins was monitored by Western blotting (B). VCL and TUBA4A served as loading controls to assess the knockdown efficiencies of all proteins relative to the control population (siC) as indicated by numbers above panels. The number of SG-positive cells (C) as well as the SG-area fraction (D) was determined via the Mica2D particle detector for ImageJ, essentially as previously described (4). Error bars indicate s.d. determined by analyzing at least 100 cells. Bars 25µm.

Figure S6. The concomitant depletion of TIA proteins and G3BP1 inhibits SG-assembly. (**A-C**) Huh7 cells transfected with indicated siRNAs (siC: control; siSG: siTIA1, siTIAR and siG3BP1) for 72h were stressed by arsenate (A; 2.5µM) or thapsigargin (T; 1µM) for 2h. SG-formation was monitored by immunostaining of indicated proteins (A). The knockdown efficiencies as well as the phosphorylation status of eIF2α were analyzed by Western blotting with indicated antibodies and quantified relative to the control population as indicated by numbers above panels using VCL and ACTB as loading controls. (B). The number of SG-containing cells was determined by counting. Error bars indicate s.d. of three independent experiments. Bars 25µm. Note that the concomitant depletion of TIA proteins and G3BP1 impairs SG-formation in a cell line and stressor independent manner (compare to Figure 1).

Figure S7. The formation of SGs remains unaffected by the single or concomitant depletion of the RBPs IGF2BP1, YB1 and HUR. (**A-H**) U2OS cells were transfected with control (siC), indicated RBP-directed siRNAs or a mixture of siRNAs targeting all three RBPs concomitantly. 72h post-transfection, cells were stressed by arsenate (2.5 mM for 1h) and SG-formation was monitored by immunostaining

with indicated antibodies (A, C, E, G). The average number of SG-positive cells was determined by counting, as indicated on the right. Errors indicate s.d. of three independent experiments. Bars 25 µm. The knockdown of indicated proteins was monitored by Western blotting and quantified relative to the control population (siC) as indicated by numbers above panels with VCL serving as loading control (B, D, F, H). Note that the formation of SGs remains unaffected by the single or concomitant depletion of the three RBPs (HUR, IGF2BP1 and YB1) analyzed.

Figure S8. FRAP (fluorescence recovery after photo-bleaching) parameters. (A) The table summarizes the exchange rates ($t_{1/2}$) and immobile fractions (in percent) averaged over the indicated number of analyzed SGs (experiments). The parameters were obtained by fitting a first order kinetic to the data. The conditions analyzed are indicated: OE-induced, SG-formation induced by transient overexpression without chemical stressor; transient/arsenate, SG-formation monitored in transiently transfected cells stressed by arsenate; stable/arsenate, SG-formation analyzed in arsenate-stressed cells stably expressing the indicated transgene. The data obtained for G3BP1, TIA1, TAIR, ZBP1, HUR or YB1 are shown in Figure 2A and B. **(B-D)** The average recovery of GFP-fluorescence in U2OS cells stably transfected with GFP-G3BP1 (B) or GFP-ZBP1 (C) was monitored shortly after SG-initiation (5-15 min arsenate treatment; green graph) or at steady state levels when SGs were completely assembled (30-60 min arsenate treatment; red graph). For ZBP1 the recovery time was increased to 90 sec to determine putative effects on the immobile fraction upon prolonged recovery time. A first order kinetic model was fitted to the data to determine the half time of fluorescence recovery ($t_{1/2\ IN}$) and immobile fractions (IF) (D). Note that the calculation of exchange rates does only apply at steady state levels, when $k_{IN} = k_{OUT}$. At the stage of SG-initiation however, we expect $k_{IN} > k_{OUT}$ since SGs are growing. Therefore only the half time of fluorescence recovery ($t_{1/2\ IN}$) can be determined.

Figure S9. The RNA-binding domains of G3BP1 are essential to promote SG-assembly. (**A**) U2OS cells were transiently (48h) or stably transfected with GFP-tagged G3BP1 wild type or mutant constructs. GFP served as negative control. Stable transfections do not represent single clones but mixed populations of approximately 100% GFP-positive cells. The number of SG-containing cells in the absence of stress was determined by immunostaining as described in Figure 4A (data not shown). Error bars indicate s.d. determined by analyzing at least a 100 transfected cells for each mutant. Note that SG-formation is only induced by the transient expression of wild type and S149-modified G3BP1 mutants. (**B**) The phosphorylation of eIF2α in stressed (+, 2.5 mM arsenate for 1h) or non-stressed cells stably expressing the indicated G3BP1 mutant proteins or GFP was analyzed by Western blotting. Note that the overexpression of G3BP1 mutant proteins does not induce or impair the phosphorylation of eIF2α.

Figure S10. SGs are dispensable for the stabilization of bulk mRNA during cellular stress. (**A**) U2OS cells were concomitantly transfected (72h) with TIA1-, TIAR- and G3BP1-directed (siSGs) or control (C) siRNAs. Where indicated cells were stressed by arsenate (2.5mM) and treated with actinomycin D (ActD, 5µM) for 2h. For each experimental condition two independent total RNA populations were analyzed by microarrays (also compare to Figure 5C-E; Affimetrix HG133plus2.0). The expression of transcripts was analyzed without normalization and background correction. The averaged transcript abundance per condition is depicted as log2-expression in box plots. ANOVA testing revealed no statistical significant differences in-between the four experimental conditions ($p > 0.01$). This indicates that bulk mRNA stabilization during cell stress is neither impaired by the depletion of TIA1/TIAR/G3BP1 proteins nor by preventing the formation of SGs. (**B**) U2OS cells were transfected with two alternative sets of TIA1-, TIAR- and G3BP1-directed siRNAs or a control siRNA for 72h. Post-transfection, cells were treated with arsenate and ActD for 1h, 2h or 4h. The change in mRNA abundance was monitored by comparative microarray analyses (Solexa HumanHT12 chips; Illumina) using untreated cells as the input control. The quantile-normalized and background corrected signals reliably detected in all three untreated conditions were averaged for both alternative sets of siRNAs. The decay-rate for each detected signal was defined as the slope of signal intensities over time (m) determined by linear regression. The m-values determined for each signal in the siSG-transfected populations are plotted over the respective m-values determined in the control (siC) populations. Note that only ~3% of all transcripts decayed more than 2-fold during cellular stress. Moreover, Pearson correlation parameters indicated in the plot, confirmed a strong coherence ($p < 0.0005$) of the determined m-values. This indicates that the impairment of SG-formation does not interfere or promote mRNA degradation during cellular stress.

Table S1: siRNAs, plasmids and oligonucleotides. The table summarizes all siRNAs with the respective sequences used in this study. If not otherwise stated all plasmids used in this study were generated by PCR on cDNA obtained from HEK293 cells using the depicted oligonucleotide sequences. PCR products were cloned into ZeroBlunt- (Life Technologies) or pGemT-Vectors (Promega), sequenced and subcloned into the described expression vectors using the restrictions enzymes shown in the table. Sequences of primer pairs used for SYBR Green I based qRT-PCR are summarized.

Table S2: Antibodies. The table summarizes all primary antibodies as well as clone numbers and providers used in this study. Secondary antibodies used for Western blotting or immunostaining were previously described (4).

1. Castets, M., Schaeffer, C., Bechara, E., Schenck, A., Khandjian, E.W., Luche, S., Moine, H., Rabilloud, T., Mandel, J.L. and Bardoni, B. (2005) FMRP interferes with the Rac1 pathway and controls actin cytoskeleton dynamics in murine fibroblasts. *Hum Mol Genet*, **14**, 835-844.
2. Kedersha, N.L., Gupta, M., Li, W., Miller, I. and Anderson, P. (1999) RNA-binding proteins TIA-1 and TIAR link the phosphorylation of eIF-2 alpha to the assembly of mammalian stress granules. *J Cell Biol*, **147**, 1431-1442.
3. Kwon, S., Zhang, Y. and Matthias, P. (2007) The deacetylase HDAC6 is a novel critical component of stress granules involved in the stress response. *Genes Dev*, **21**, 3381-3394.
4. Stohr, N., Kohn, M., Lederer, M., Glass, M., Reinke, C., Singer, R.H. and Huttelmaier, S. (2012) IGF2BP1 promotes cell migration by regulating MK5 and PTEN signaling. *Genes Dev*, **26**, 176-189.
